# An in situ study of abyssal turbidity-current sediment plumes generated by a deep seabed polymetallic nodule mining preprototype collector vehicle

**DOI:** 10.1126/sciadv.abn1219

**Published:** 2022-09-21

**Authors:** Carlos Muñoz-Royo, Raphael Ouillon, Souha El Mousadik, Matthew H. Alford, Thomas Peacock

**Affiliations:** ^1^ Department of Mechanical Engineering, Massachusetts Institute of Technology, Cambridge, MA, USA.; ^2^ Scripps Institution of Oceanography, University of California, La Jolla, CA, USA.

## Abstract

An in situ study to investigate the dynamics of sediment plumes near the release from a deep seabed polymetallic nodule mining preprototype collector vehicle was conducted in the Clarion Clipperton Zone in the Pacific Ocean 4500-m deep. The experiments reveal that the excess density of the released sediment-laden water leads to a low-lying, laterally spreading turbidity current. At the time of measurement, 2 to 8% of the sediment mass were detected 2 m or higher above the seabed and were not observed to settle over several hours, with the remaining 92 to 98% below 2 m and some fraction of that locally deposited. Our results suggest that turbidity current dynamics sets the fraction of sediment remaining suspended and the scale of the subsequent ambient sediment plume. The implications of this process, which is characteristically overlooked in previous modeling efforts, are substantial for plume modeling that will lie at the heart of environmental impact statements for regulatory consideration.

## Teaser

In situ deep-sea nodule mining monitoring shows that turbidity currents set sediment plume evolution, deposition, and suspension.

## INTRODUCTION

**Fig. 1.**
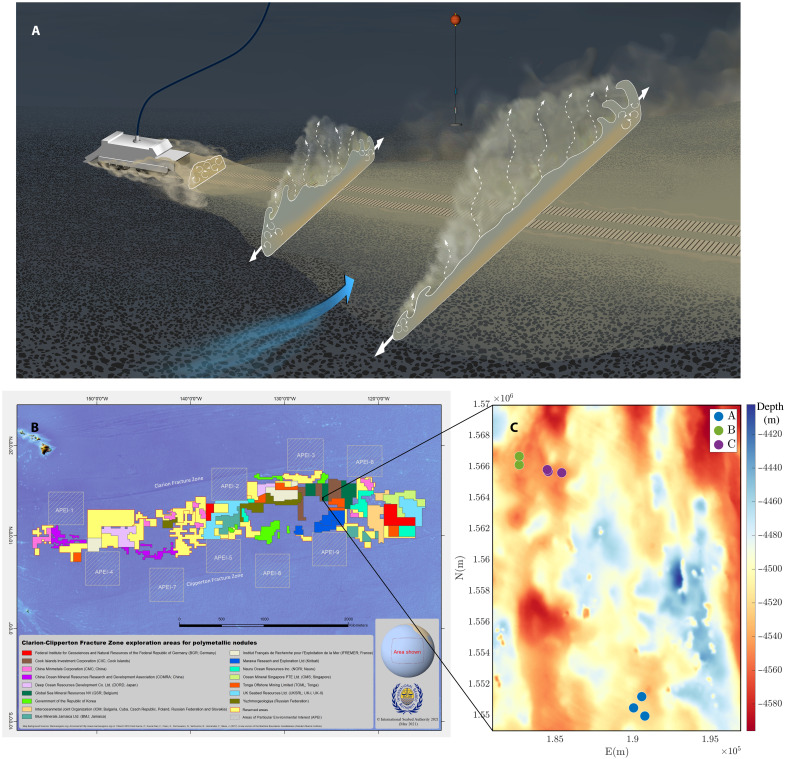
Plume illustration and field study location map. (**A**) Illustration of the plume spreading behind a collector vehicle as it picks up polymetallic nodules. Three cross sections of the plume are shown to illustrate the evolution of the plume. In the first cross section, closest to the vehicle, the high turbulence of the vehicle wake mixes the sediment. In the middle cross section, the plume is spreading under its own buoyancy as a turbidity current as indicated by the thick white arrows. At the same time, fine sediment particles are detraining from the body of the turbidity current as indicated by the dashed arrows. In the third cross section, the turbidity current is still spreading under its own buoyancy, but the effect of the cross flow (blue arrow) leads to a more evident asymmetry and a lower and taller fronts spreading in the opposite and same directions as the cross flow, respectively. (**B**) Map of the CCZ exploration areas (source: International Seabed Authority, 2021) and (**C**) zoomed-in view of the location of the A, B, and C experiment sites superimposed to local bathymetry map (*30*). The axes are in Universal Transverse Mercator (UTM) zone 10 coordinates.

With an increasing international focus on the opportunities and costs of deep seabed polymetallic nodule mining (*1*), a pressing matter to be resolved is the scale of the benthic sediment plume (hereinafter referred to as “sediment plume” or “plume”) that would be generated by these activities (*2*). Proposed operations will use a collector vehicle with a pick up mechanism, which may be hydraulic or mechanical, that will remove both nodules and the upper layer of the sediment as the vehicle maneuvers on the abyssal seabed (Fig. 1A). The desired polymetallic nodules would be separated from the sediment within the body of the collector vehicle, the nodules being transported to a surface operation vessel, and most of the sediment being discharged in the vicinity of the collector. A fundamental question regarding the scale of the sediment plume is what fraction of the sediment disturbed by a collector vehicle would be deposited locally at the mining site versus what fraction of sediment would be transported away by background currents and with what characteristics (e.g., vertical concentration profile, sediment particle size, and settling speed characteristics). All modeling of plume transport away from a mining site, which will form the basis of estimates of indirect environmental impact for deep seabed mining (i.e., direct impact will be caused by the collector tracks and the removal of the nodules and sediment), requires this information as a critical input to make predictions (*3*, *4*); any errors in model assumptions about these local initial conditions can have profound implications for predictions of distant plume transport. A thorough understanding of the initial form of collector plumes is also the foundation for designing approaches to polymetallic nodule mining that, to the best of their abilities, can mitigate the associated environmental impacts.

Until this study, no in situ data are available to develop physical understanding and quantification of the nature of sediment plumes near a collector vehicle. The National Oceanic and Atmospheric Administration (NOAA) Deep Ocean Mining Environmental Study (DOMES) comprised a towed collector vehicle operating at a depth of 5000 m in the Clarion Clipperton Zone (CCZ) (*5*). Heavy (centimeter-scale) resedimentation was observed for several meters either side of the collector track, with lesser coverage of the nodule field out to around 100 m. Several benthic impact experiments (BIEs) have sought to mimic and study the sediment disturbance created by a deep seabed polymetallic nodule mining operation. The Disturbance and Recolonization (DISCOL) experiment was conducted using a plough-harrow in a 3.7-km-diameter circular region of the Peru Basin, at a depth of around 4150 m (*6*). Photography and videography recorded activities during the experiment, and the site was revisited several times, with a substantial reanalysis of the track patterns (*7*). Sediment core analysis determined deposition thicknesses to range from 1 to 2 mm at some locations to 10 to 30 mm at others. Subsequently, the NOAA-initiated BIEs (*8*) observed heavy resedimentation, with 1 to 2 cm of coverage 50 m away from the collector tracks, dropping off rapidly at distances of 300 to 400 m. Of the aforementioned studies, only the DOMES experiment used technology intended for polymetallic nodule collection, whereas the approaches used for DISCOL (plough-harrow) and BIE (vertical tube pump) likely initiated a somewhat different form of disturbance. None of the aforementioned studies, however, comprised any monitoring of the collector plume in the immediate vicinity of a moving collector vehicle.

Several modeling efforts have attempted to simulate far-field sediment plume dispersal from test- or commercial-scale deep seabed polymetallic nodule mining operations. Two recent studies looked into the potential influence of remotely generated eddies (*3*) and sediment aggregation (*4*). For all such models, however, an inherent challenge is that the finest resolved scale of the simulation is coarse compared to the scale of a collector vehicle and its wake structure. Hence, broad assumptions have to be made about the initial form of the sediment disturbance created by a collector vehicle, which, in turn, limit the skill of far-field predictions. A typical approach has been to assume a certain mass pick-up rate of sediment based on the expected operational parameters of a collector (e.g., span, speed, and thickness of sediment layer collected) and to distribute this sediment load uniformly across the lowest resolved levels of the numerical model, which have been on the order of 5 to 10 m (*3*, *4*). These assumptions are not well founded, however, as key fluid dynamical processes in the wake of the collector, operating on much smaller scales than has been numerically resolved, are already known to give rise to heavy local deposition (*8*, *9*). For example, the observations in previous BIEs using a plough-harrow, which did not fully disaggregate the cohesive sediment nor release the sediment notably above the seabed, led to a conclusion that around 90% of the sediment settles within 10 to 100 m of a collector track and around 10% of the sediment remain in suspension to be carried away by background ocean currents (*7*). However, key observations such as this, which could readily affect plume predictions by an order of magnitude, have yet to be incorporated into any collector plume modeling. Thus, a key step forward is to develop modeling approaches that are informed by observations and/or physical understanding of processes in the vicinity of a collector vehicle.

A collector will likely discharge sediment into a wake region of notable turbulence generated by the collector motion (see Fig. 1A). The intrinsically turbulent sediment-laden discharge is expected to have some momentum and, furthermore, be denser than ambient fluid, giving it a propensity to sink toward the seabed under the effect of gravity. A dimensional analysis of the three competing physical processes suggests that most likely the wake turbulence will dominate over both the momentum and the negative buoyancy of the discharged fluid (*10*). More formally, the balance between the inertial forces of the turbulent wake and the negative buoyancy forces of the discharge can be characterized by a Froude number, which is the ratio of the characteristic wake velocity to the discharge buoyancy velocity, defined as $Fr=\frac{U_{c}}{\sqrt{g\prime h}}$, where *U*_c_ is the speed of the collector, *h* is the vertical extent of the discharge diffuser, and $g\prime =g\frac{\rho_{o}-\rho_{a}}{\rho_{a}}$ is the reduced gravity, *g* being gravitational acceleration and ρ_o_ and ρ_a_ the densities of the outflow and ambient fluid, respectively. The density of the sediment-laden outflow is ρ_o_ = ϕρ_p_ + (1 − ϕ)ρ_a_, where ρ_p_ is the density of individual particles and ϕ is the volume fraction of particles, which, in turn, is estimated from the mass flux of discharged sediment $\overset{\cdot }{m}$ and the volume flux at the diffuser *Q* via $\phi =\frac{\overset{\cdot }{m}}{Q\rho_{p}}$. *Fr* > 1 at the discharge indicates that the wake turbulence initially dominates over the negative buoyancy and vice versa when *Fr* < 1. For the preprototype collector vehicle that is the focus of this study, *U*_c_ ∼ 0.3 m/s, *h* ∼ 30 cm, *Q* ∼ 1 *m*^3^/s, $\overset{\cdot }{m}∼10$ kg/s, ρ_p_ ≈ 2600 kg/m^3^, and ρ_a_ ≈ 1030 kg/m^3^, such that *Fr* ≈ 2.3 > 1, suggesting that mixing will initially play a substantial role in the wake immediately after discharge.

In the limit of large Froude number (i.e., very strong turbulent mixing behind the collector), the concentration of sediment in the water column behind a collector after discharge will be reasonably approximated by $\frac{\overset{\cdot }{m}}{U_{c}A}$, where *A* is the cross-sectional area of the wake that will be comparable to the vehicle cross-sectional area (*11*). For the investigated collector vehicle with *A* ∼ 16 m^2^, the concentration of sediment in the wake will therefore be around 2 kg/m^3^, which is more than sufficient to form a turbidity current (*12*) in which the sediment-laden discharge propagates under the influence of its own negative buoyancy (see the “Discharge characterization” section in Methods). The presence of a turbidity current in the wake of a polymetallic nodule collector has been hinted at in previous works [e.g., (*13*–*16*)] and is consistent with reports of heavy redeposition close to disturbance tracks in BIEs (*7*–*9*). Most recently, numerical modeling and laboratory experiments (*10*) have shown that the ratio of collector speed to the appropriate buoyancy velocity (i.e., the velocity that results from the release of dense fluid in a relatively lighter fluid; see the “Turbidity current features” section in Results) controls the dynamics of such a turbidity current. Above a critical value of this ratio, the turbidity current reaches a steady state in the reference frame of the moving vehicle, and it propagates mainly in the direction normal to that of the collector, forming a wedge shape behind the collector (Fig. 1A). A recent modeling effort (*17*) did not consider the role of the vehicle’s turbulent wake and three-dimensional effects in the vicinity of the collector vehicle, which this study shows to be vital considerations. Flow conditions in the deep ocean are not quiescent, however, and a collector plume will interact with background currents. Although the role of cross flows on turbidity currents has been investigated for some canonical configurations (*18*), the impact of background currents on a turbidity current associated with a collector plume is still unknown. Confirmation and quantification of turbidity current dynamics in the wake of a collector and in the presence of deep ocean flow conditions would be a major advance in understanding and modeling deep seabed polymetallic nodule mining sediment plumes.

In April to May 2021, the Belgian contractor Global Sea Mineral Resources NV (GSR) performed the first preprototype nodule collector vehicle (hereinafter referred to as “collector”) trials in the abyssal Pacific Ocean since the late 1970’s, at a depth of 4500 m (Fig. 1, B and C). The collector was heavily instrumented with sensors that enabled interrogation of the sediment plume in the immediate vicinity of the vehicle. A series of custom-designed operational maneuvers, termed “selfies” and “drive-bys,” were performed at three different sites to enable the nodule collector to intersect the collector plume close to the original disturbance location and measure its properties at several instances in its evolution history. Here, we present the results of this unique experiment, which confirms and makes direct measurements of the turbidity current phase of a sediment plume associated with deep seabed polymetallic nodule mining activities. These results lay a foundation for improved modeling of the far-field evolution of the plume and provide highly valuable physical insight and data that can be used to initiate simulations of commercial-scale deep seabed polymetallic nodule mining operations.

## RESULTS

### Turbidity current features

**Fig. 2.**
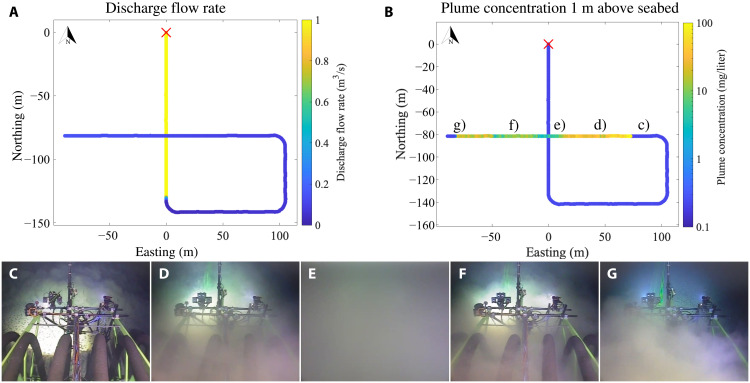
Selfie A3 top view and front-camera images. (**A** and **B**) Top view of the collector position during selfie A3. The colormap represents (A) the discharge flow rate in m^3^/s and (B) the plume sediment concentration above ambient levels in mg/l measured by the STM 1 m above the seabed. The selfie maneuver starts in the location indicated by the red cross, which corresponds to the origin of the coordinate system. (**C** to **G**) Snapshots from the top-mounted camera on the collector vehicle at the locations identified in (B).

To investigate the nature of the sediment plume in the wake of the collector vehicle, a maneuver termed as a selfie was devised. In this maneuver, the collector drove an ∼100-m track collecting nodules before turning off its collection system, conducting three 90^∘^ turns with intervening traverses, and proceeding to drive back perpendicularly across the collection track (Fig. 2A and fig. S1), thereby encountering its own plume (Fig. 2B). These operations were designed so as to encounter the plume in its turbidity current regime, if present, and while traveling in a direction perpendicular to the expected propagation direction (see the “Discharge characterization” section in Methods). As expected, the collector only encountered the plume during the last segment of the maneuver, and each selfie was iteratively optimized to encounter the plume at an earlier time in its life cycle than the previous selfie. The design of the maneuver, the collector instrumentation, and the list of maneuvers performed are detailed in the “Selfie experiments” section in Methods.

Snapshots from a forward-facing camera mounted on the top of the collector, 4 m above the seabed, are shown in Fig. 2 (C to G) at five key times: (i) immediately before the plume is encountered (Fig. 2C), (ii) between the first encountered front of the plume and the collection track (Fig. 2D), (iii) when the collector crosses the collection track (Fig. 2E), (iv) between the collection track and the second encountered front of the plume (Fig. 2F), and last, (v) once the collector is exiting the plume (Fig. 2G). The image in Fig. 2C shows the collector driving through clear water, with polymetallic nodules observable on the seabed in the foreground and the sharp front of the oncoming plume in the background. In Fig. 2D, once the collector has entered the plume, the mechanical structure at the front of the collector is visible, but not the seabed, showing that the plume is low-lying. In Fig. 2E, which occurs when the collector is driving over its previous collection track, the camera is surrounded by sediment, and there is no visibility. In Fig. 2F, once again, the collector is among a low-lying plume. Last, in Fig. 2G, the collector is exiting the plume via a second, very sharp front similar to that it encountered when entering the plume; the mechanical structure at the front of the collector is in clear water, and polymetallic nodules are visible in the background, but the sediment is still seen in the foreground between the camera and the front of the vehicle. Qualitatively, this sequence of events is consistent with a turbidity current spreading perpendicularly away from the collector track. As we advocate later, the higher elevation of sediment in Fig. 2E encountered when crossing the collector track is seemingly due to mixing by the turbulent wake of the vehicle during nodule collection, leaving suspended, detrained sediment up to a height roughly comparable to that of the vehicle.

**Fig. 3.**
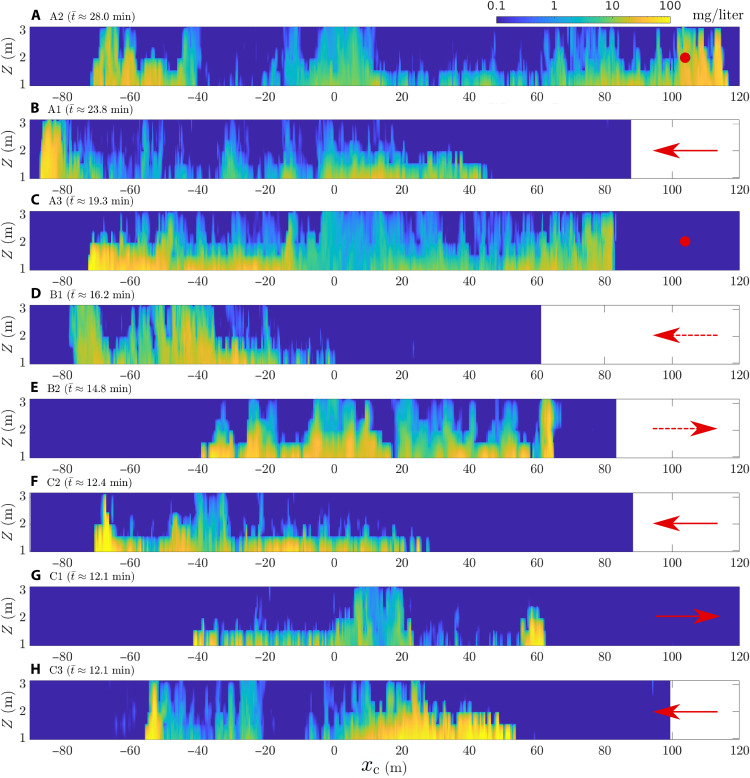
Sediment plume concentration vertical profiles measured during selfie experiments. Colorplot of vertical profiles of sediment plume concentration above ambient levels as a function of the distance along the last leg of the selfie tracks *x_c_*, with *x_c_* = 0 representing crossing the first leg of the maneuver when the collection system was running. The selfies in (**A**) to (**H**) are presented in order of decreasing values of $\bar{t}$. The sediment concentration is linearly interpolated in between the STMs in the vertical direction. The arrows and dots indicate the presence or absence of a crossflow, respectively; the arrow length does not represent magnitude. Solid arrows correspond to measured current heading using ADCP data from a nearby mooring, and dashed arrows are inferred on the basis of ADCP data available during adjacent time windows (i.e., assuming persistent current directions). The lowest concentration in the logarithmic colormap (0.1 mg/liter) is one order of magnitude above the noise level of the instrument.

Figure 3 presents the vertical profile of the sediment concentration above ambient levels (hereinafter simply referred to as plume concentration) measured by the front-mounted Seapoint Turbidity Meters (STMs) (see Methods) for the eight selfies that were performed; three at site A, two at site B, and three at site C (see Fig. 1). The operational parameters of the selfies are summarized in Table 1. A top view of each selfie is presented in fig. S2. Given that the discharge parameters were varied for three of the eight selfies and that the trajectory itself was iteratively improved from one selfie to the next to intersect the plume earlier in its turbidity current phase, the data contain a wealth of spatiotemporal information. We plot the profiles for the last leg of the selfie maneuver as a function of the distance to the collection track using the coordinate *x*_c_, defined as the position of the collector on the *x* axis of the selfie coordinate system (see Methods). As the maneuver was different for each selfie, we indicate the time $\bar{t}$ that it took for the collector to execute the loop maneuver, i.e., the difference between the first and second times it passed through the intersection point on the collection track (see the “Selfie experiments” section in Methods); this value only depends on the maneuver design and the collector speed, but it is a good approximation of how long the turbidity current had to propagate before being intersected by the collector. In Fig. 3 (A to H), the selfies are ordered by decreasing value of $\bar{t}$, i.e., ordered by increasingly short maneuvers and propagation times. The sediment is rarely observed at the uppermost STM, suggesting that the turbidity current is typically less than 3-m tall (the lowest STM was at 1 m). In most selfies, the sediment concentration is highly heterogeneous, both in the vertical direction and along its propagation direction perpendicular to the collector tracks. To interpret the data in these figures, however, it is important to remember that the turbidity current is a transient process, such that the vertical profiles in Fig. 3 (A to H) are not snapshots at a particular time but rather a sampling of the current at different space-time locations; more specifically, the turbidity current is sampled at later and later times in its evolution as *x*_c_ increases.

**Table 1.** Summary table of the eight selfie and two drive-by experiments conducted during the field studies. In the case of the selfies, the length of segment *L*_3_ was reduced, and the collector vehicle velocity (*U*_c_) was increased to reduce $\bar{t}$ and intersect the turbidity current earlier. The slope and downslope direction were obtained from the 50-m resolution bathymetry available from the area (*30*).

**Selfie**	***L*_3_ (m)**	***U*_c_ (m/s)**	**$\bar{t}$ (min)**	**Initial heading (°T)**	**Slope (°)**	**Downslope direction (°T)**
A1*	130	0.23	23.8	269	<1	–
A2	170	0.25	28.0	359	∼1–1.5	∼45
A3	100	0.28	19.3	179	<0.5	–
B1^†^	100	0.36	16.2	271	∼1	∼90
B2	100	0.30	14.8	359	<1.2	–
C1	90	0.39	12.1	25	<1	∼225
C2	90	0.39	12.4	274	<1	∼45
C3	60	0.40	12.1	184	<1	–
**Drive-by**	***D* (m)**	***U*_c_ (m/s)**	**Mooring**	**Initial heading (°T)**	**Slope (°)**	**Downslope direction (°T)**
DB1*	100	0.28	MA	89	<0.5	–
DB2	50	0.32	MC	93	∼1	∼225
DB3*	100	0.27	MA	269	<0.5	–
DB4	100	0.26	MA	359	<0.5	–

*Selfie experiment A1 and drive-by experiments DB1 and DB3 were conducted with two of the four pumps of the collection system at providing 50% of the standard flow rate.

†Selfie experiment B1 was conducted with the collection system turned off.

In general, distinctive turbidity current features can be identified in most of the STM selfie datasets. Well-formed heads in which the concentration of sediment is greatest and reaches higher elevations above the seabed are readily observed in the initial front of selfies A1, A2, A3, B1, C2, and C3, as well as in the second front of selfies A2, A3, B2, and C1. In between these heads, a thinner body—typically reaching 1 to 2 m above the seabed and displaying lower sediment concentrations—is observed. These features are characteristic of a turbidity current propagating under its own buoyancy and leaving a trailing wake, as is widely reported in constant volume lock-release experiments [e.g., (*19*, *20*)]. In some of the selfies, no well-defined head is encountered at the entry (B2 and C1) or exit (A1, B1, C2, and C3) fronts; instead, the sediment is found at similar heights as the body of the current, albeit in often higher concentrations. Last, most of the selfies show a region of elevated concentrations at greater height above the seabed in the vicinity of *x*_c_ = 0 (i.e., when the collector crosses the collection track). This is particularly visible in A2 at *x*_c_ ≈ 5 m, in A3 at *x*_c_ ≈ 0 m, in B1 at *x*_c_ ≈ −45 m, in B2 at *x*_c_ ≈ 0 m, in C2 at *x*_c_ ≈ −35 m, in C1 at *x*_c_ ≈ 15 m, and, to an extent, in C3 at *x*_c_ ≈ −30 m. Our analysis in the section entitled “The central patch” shows that this disturbance is not from a turbidity current but rather is sediment maintained in suspension by turbulence in the wake behind the collector during the collection process.

A general observation is that the plume spreads further away from the tracks when it is intersected at later times (i.e., for larger values of $\bar{t}$) (see Fig. 3). Combined with the observations of a distinct front on both sides of the tracks in the vertical profiles of concentration, this suggests that the turbidity current retains a substantial amount of momentum and suspended sediment even after propagation times of over 20 min. The distance between the two fronts at a given time *t*, which characterizes the total breadth of the current, is not fully known, as a turbidity current is a transient process with a time-varying propagation speed, and Fig. 3 is not an instantaneous snapshot. With some appreciation of turbidity current dynamics in hand, however, knowing the times *t*_1_ and *t*_2_ at which the collector encounters the first and second fronts, we can approximate the distance between the fronts at the average time $\frac{t_{1}+t_{2}}{2}$ as

$$L_{f}(\frac{t_{1}+t_{2}}{2})\approx x_{f}^{2}(t_{2})-x_{f}^{1}(t_{1})$$ (1)

where $x_{f}^{1}$ and $x_{f}^{2}$ are the positions of the first and second fronts, respectively (see the “Selfie experiments” section in Methods). In making this approximation, it is important to recognize the role of background currents, particularly crossflows, which might contribute significantly to the transport of sediment. For now, we assume that any crossflow component of the background flow simply augments the sediment transport velocity by that velocity. In reality, a crossflow interacts hydrodynamically in a complex fashion with the turbidity current, altering its shape and sediment distribution; as a result, only a fraction of the momentum of the background current may be transferred to the turbidity current [see (*18*) and the discussion on the role of crossflows in the “Role of background currents” section]. We thus consider that the error in Eq. 1 can be caused by an advection term of ±6 cm/s, which is the maximum observed current speed during the selfie experiments. We account for the potential scale of this error using Taylor series expansion (see the “Estimating the distance between the two fronts” section in Methods).

**Fig. 4.**
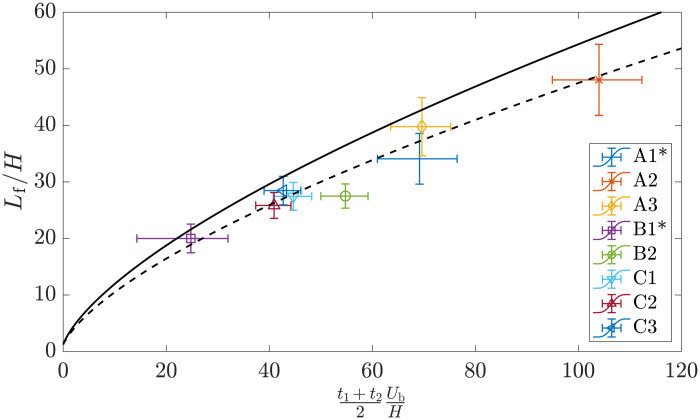
Distance between turbidity current fronts for all selfie experiments. Distance between the two fronts at time $\frac{t_{1}+t_{2}}{2}$, approximated as $L_{f}=x_{f}^{2}(t_{2})-x_{f}^{1}(t_{1})$ for all selfies. The results are presented in their nondimensional form, scaled by initial release height *H* and buoyancy velocity *U*_b_. The buoyancy velocity is computed assuming a discharge of 12 ± 3 kg/s of sediment for all selfies except A1 and B1, which ran with two active pumps and zero active pump, respectively, and for which, we assume a discharge of 9 ± 2 and 3 ± 2 kg/s of sediment (*see the “Discharge characterization” section in Methods). The vertical bars account for the margin of error assuming a crossflow advection of *U*_⊥_ = ± 6 cm/s (see main text for discussion). The full black line corresponds to the solution to the box model approximation (see the “Box model” section in Methods) assuming a discharge of 12 kg/s, while the dashed black line corresponds to the box model solution assuming a discharge of 8 kg/s.

The approximated distance *L_f_* is compared to a box model calculation (see the “Box model” section in Methods) for each selfie in Fig. 4. All selfies but two were run with all four collection system pumps active, resulting in a discharge of $\overset{\cdot }{m}\approx 12\pm 3$ kg/s of sediment (see the “Discharge characterization” section in Methods for the estimation of sediment discharge rates). During selfie A1 and B1, two and zero collection pumps were active, respectively, giving rise to estimated discharge mass flow rates of 9 ± 2 and 3 ± 2 kg/s, respectively. The selfie-specific buoyancy velocity $U_{b}=\sqrt{g\frac{\overset{\cdot }{m}}{\sqrt{2}U_{c}H}\frac{\rho_{p}-\rho_{w}}{\rho_{w}\rho_{p}}}$ (see the “Discharge characterization” section in Methods) is used to nondimensionalize time in Fig. 4 such that all selfies can be compared to a single box model calculation, with good qualitative agreement between the distance predicted by the box model and the data from selfies. The results are additionally compared to the box model approximation assuming that only 8 of the 12 kg/s discharged became part of the gravity current (dashed line), with improved agreement. Several reasons might explain this improved agreement when assuming a slightly weaker discharge, including (i) measurement errors that result in an overestimation of the discharge mass flow rate, (ii) inaccuracy of the box model approximation and its parameterization, (iii) rapid settling of large/flocculated particles that do not become part of the gravity current after being discharged, (iv) detrainment of sediment in the wake that does not become part of the gravity current, and (v) settling/deposition dynamics that reduce the negative buoyancy of the current as it spreads.

### Role of background currents

#### Front propagation and geometry of the head

The turbidity currents released over the course of the different selfies, described in the “Turbidity current features” section, propagated in a nonquiescent ambient. The role played by crossflows on the propagation of gravity currents has been investigated theoretically and experimentally in the case of rectilinear currents for both a constant volume (*18*) and constant flux (*21*) release. In the case of the collector discharge, the turbidity current can be understood as a constant volume lock release in the plane normal to the direction of motion of the collector (*10*). It follows that we expect the component of the background current that is parallel with the direction of propagation of the turbidity current to act similarly as investigated in (*18*), which is increasing the height and speed of the along-current head of the turbidity current and decreasing the height and speed of the against-current head of the turbidity current.

While background current data are not available for all the selfies (see the “Background currents” section in the Supplementary Materials), the available data indicate that the background currents were predominantly southward during the A experiments and southwestward during the C experiments (no current data are available during the B experiments). Because all selfies had the collection track aligned with either the north-south axis or the west-east axis, we anticipate that, in some of the selfies, the released turbidity current experienced a weak to moderate component of the background current as a crossflow, while in others, the turbidity current experienced close to the full magnitude of the background current as a crossflow. When the Acoustic Doppler Current Profiler (ADCP) data are available, we project the background current into the reference frame of the selfies (see details in the Methods-Selfie Experiments section) to determine the magnitude and sign of the background current that acts as a crossflow on the turbidity current. Quantitatively, this component is given by

$$U_{\text{cf}}=u_{e}\cdot e_{x}$$ (2)

where **u**_**e**_ is the background current in vector form and **e**_**x**_ is the unit vector defined positive on the *x* axis of the coordinate system in the case of selfies. The component of the current that acts parallel to the tracks and thus normal to the crossflow is referred to as the spanwise component.

The ADCP data available for selfies A1 and A2 revealed a clear southward heading, with headings of 179^∘^ true (T; i.e., respect to the geographic north) and 187^∘^T, respectively (see table S1 and fig. S3). The orientation of A1 (refer to fig. S1) is such that the background current acted as a crossflow, oriented in the direction opposed to the *x* axis of the selfie, with a negligible spanwise (i.e., along track) component; for illustration purposes, the crossflow component is added to Fig. 3 as a red arrow or dot, indicating the direction or the absence of a crossflow component, respectively (the arrow length does not represent magnitude). As a result of this substantial crossflow and based on the findings of (*18*), we expect that the leftward front to propagate more rapidly than the rightward front and the leftward head of the current to be taller than the rightward head. Both phenomena are clearly observable in Fig. 3B, with the leftward front reaching heights of 3 m and traveling further from the tracks than the rightward front (although it was intersected later in the maneuver), with a head reaching only 1.5 m above the seabed. This markedly contrasts with selfie A2, the orientation of which is such that the background current acted as a spanwise component, with a negligible crossflow component. In Fig. 3A, we see that the A2 vertical profiles of the sediment concentration are more symmetric around the tracks than during A1, with both turbidity current heads reaching similar heights and displaying similar shapes and sediment concentration. The rightward front reaches a distance from the track that is larger than the leftward front, which is expected in the absence of a crossflow as the rightward front is intersected later, and has therefore more time to propagate than the leftward front. While no ADCP data are available for A3, consistency in the current heading throughout the A experiments suggests that A3 likewise experienced a predominantly southward current, which would result in a predominantly spanwise component of the current, and a negligible crossflow component. Once again, this observation is highly consistent with the vertical profile of concentration during A2 (see Fig. 3), which shows strong symmetry of the current on either side of the tracks.

During the C3 experiment, current was southwestward, *U*_e_ ≈ 6.3 cm/s and heading of 217^∘^T during and following C3 (see table S1 and fig. S3). The orientation of the C3 selfie is such that the background current contributed to both a spanwise and crossflow component of magnitude 3.8 cm/s, oriented in the negative direction along the selfie’s *x* axis. As for A1, the turbidity currents of C3 are clearly affected by the crossflow (see Fig. 3H), with the leftward front propagating in the same direction as the crossflow component, leading to a fast propagating front with a sharply defined, taller head. The rightward front, although intersected at later times, propagated over a shorter distance from the tracks, with an elongated and lower head. Although ADCP data are not available for C1 and C2, current data for 48 hours during and after C3 indicate a persistent southwestward current, and both vertical profiles of the turbidity currents are consistent with this. The orientation of C2 is such that it experienced a similar crossflow as C3, which is consistent with observations of a stronger leftward propagating front with a taller head and a much slower, thinner head at the rightward front. In C1, the turbidity current experienced a slightly larger crossflow component (Eq. 2), oriented positively along the *x* axis of the selfie. Consequently, the vertical profiles of concentration reveal a much sharply defined head at the front in the direction aligned with the crossflow, this time the rightward front, and a much slower and thinner head at the front that propagates in the direction opposed to the crossflow, this time the leftward front.

Last, the vertical profiles of concentration in Fig. 3 suggest that B1, which had the weakest discharge of all selfies with all pump heads turned off, experienced a strong crossflow component oriented negatively along the selfie’s *x* axis. Given the orientation of B1, this suggests a strong southward component of the background current, consistent with both the A and C experiments, the latter of which was close in time and space. Interpretation of the B2 selfie is more ambiguous. The rightward front reaches further from the tracks than the leftward front, but this is expected even in the absence of a crossflow component, as the rightward front is always encountered later than the leftward front in a selfie maneuver. However, the current head at the leftward front is thinner than the head at the rightward front, suggesting a nonnegligible crossflow in the positive *x* direction. Given the orientation of selfie B2, the observations suggest that the background current has a nonnegligible westward component, although likely smaller than the southward component.

**Fig. 5.**
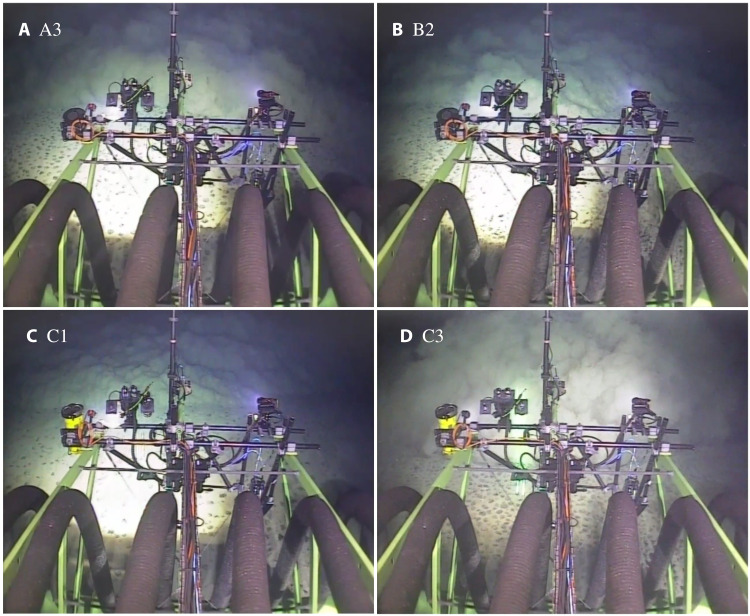
Turbidity current head of the first front during four different selfie maneuvers. During (**A**) A3, the first front experienced a negligible crossflow component of the background current. During (**B**) B2 and (**C**) C1, the first front experienced a negative crossflow component, resulting in a thinner head. During (**D**) C3, the first front experienced a positive crossflow component, resulting in a taller, more concentrated head.

Snapshots obtained from video footage immediately before entering the first front of the turbidity current provide direct visual confirmation of the role of the crossflow component of the background current on the head of the turbidity current (see Fig. 5). During selfie A3, the crossflow component of the background component was negligible, and the head of the first front encountered is clearly defined as a sharp, turbulent sediment front. The head of the first front of the B2 and C1selfies, however, whose spreading was opposed by the crossflow component, are evidently thinner and with smaller turbulent features; the very edge of the heads of these currents is strongly inclined instead of vertical, further highlighting the role of the crossflow on the head geometry and hydrodynamics. Last, the head of the first front encountered during the C3 selfie, for which the crossflow augmented the propagation of the front, is markedly taller than the other selfies and displays larger turbulent features.

#### The central patch

In addition to the turbidity current behavior, a taller structure is found between fronts in a number of selfies. This structure is hypothesized to result from turbulence in the wake of the collector creating a region of particle-laden fluid that does not become part of the turbidity current. In the following, we refer to this region as the “central patch” of sediment. Of note is that while selfie B1 had the weakest sediment discharge the vertical profiles of concentration reveal the strongest instance of a central patch between the fronts (see Fig. 3D), which suggests that the amount of sediment in this feature depends on the interaction between wake turbulence and the stabilizing effect of the negatively buoyant discharge (i.e., on the Froude number of the collector discharge). It also suggests that a fraction of the discharged sediment does not become part of the turbidity current. We note that the vertical form of this central patch is quite homogeneous, suggesting that the background currents were vertically uniform between 1 and 3 m in height.

The center of mass of a turbidity current subjected to a crossflow is thought to be translated at a fraction (∼60%) of the mean background velocity (*18*). While it is expected that any crossflow component of the background current will also advect the central patch along the selfie’s *x* axis, because it is not part of the turbidity current, this may be in a manner different to the influence of the background flow on the turbidity currents. In both experiments A2 and A3, for which the background current yielded a negligible crossflow component, the central patch is observed to sit close to the tracks (*x*_c_ ≈ 0 m), which is consistent with the observed symmetry of the fronts on either side of the tracks. During selfie C3, however, the central patch was encountered after approximately 12 min at *x*_c_ ≈ −30 m, suggesting an advection velocity along the *x* axis of approximately 4 cm/s, which is in excellent agreement with an established crossflow component of 3.8 cm/s measured nearby by an ADCP. For B1 and C2, the central patch is observed at a distance of *x*_c_ ≈ −40 m and *x*_c_ ≈ 35 m, respectively, at times *t* ≈ 15 min and *t* ≈ 11 min after release, respectively. This corresponds to advection velocities of 4.4 and 5.3 cm/s, respectively. While ADCP data are not available for these particular experiments, these values agree well with the current velocity magnitudes measured at other times in the same area (see table S1 and fig. S3) and are significantly higher than ∼60% of the background velocity that is the case for turbidity currents (*18*). Hence, this central patch appears to be advected with a velocity equal to the whole crossflow component and is a feature of the collector plume that is distinct from the buoyancy-driven turbidity currents that spread to either side of the tracks.

#### Drive-bys

In addition to the eight selfies, four drive-by maneuvers were conducted (DB1, DB2, DB3, and DB4; see the “Drive-by experiments” section in Methods and fig. S1). In DB1 and DB2, the collector drove eastward along a straight line 100 m north of the mooring MA and 50 m north of the mooring MC, respectively. In DB3, the collector drove westward 100 m south of mooring MA, and in DB4, the collector drove northward 100 m west of mooring MA. Because drive-bys DB1 and DB2 were carried out north of the mooring and in the presence of southward background currents (see Table 1), it is expected that the north-propagating turbidity current from the drive-by will slow down to a velocity below the background current velocity, at which point it will become advected to the south and eventually pass by the mooring. Thus, measuring sediment concentration at the mooring should allow to observe not only the south-propagating front but also the (initially) north-propagating one. In the case of drive-by DB3, which was conducted south of the mooring with a southward background current, no turbidity signal was detected at the mooring because of the southward current (see the “Runout length” section in Results). DB4 did not result in a usable turbidity signal because of the southward current (see more details in the “Drive-by experiments” section in Methods).

**Fig. 6.**
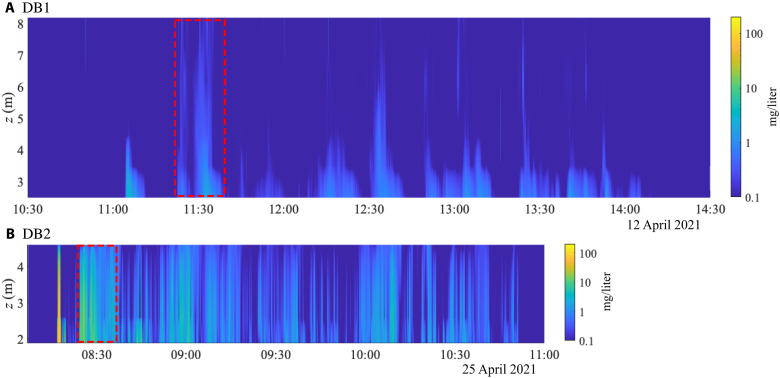
Interpolated vertical profiles of plume sediment concentrations above ambient levels from mooring-mounted STMs. Vertical profiles measured during the (**A**) DB1 drive-by and (**B**) DB2 drive-by. Both drive-by tracks were located north of the mooring, in the presence of a southward ocean current. At late times in the propagation of the turbidity current, the background current becomes larger than the velocity of the current front, such that even the north-propagating current front eventually becomes advected south and passes by the mooring, resulting in a continuous but finite observation time window of the plume at the mooring. The central patch (red rectangle) of sediment is the first tall structure to pass through the mooring after the first front.

The timing of each plumes’ passage by the moorings is captured by a vertical array of STMs mounted on the mooring (see the moorings layout in fig. S4) at 2.5, 3.5, 4.5, 6.2, and 8.2 m above the seabed in the case of mooring MA and at 1.9, 2.6, and 4.6 m above the seabed in the case of mooring MC. Figure 6 shows the vertical profiles of concentration interpolated along the vertical axis for both drive-bys, where the first plume signal is identified as the first front of the turbidity current, and the last plume signal as the second front of the turbidity current, passing by the mooring in the direction of the crossflow, which is opposed to its direction of initial propagation. Relatively soon after the first front passes through the mooring during both drive-bys, the central patch (identified by the red dashed lines in Fig. 6) crosses the mooring. Backscatter intensity data from the mooring-mounted ADCP (see fig. S5) reveal that the central patch is characterized by the presence of sediment at heights of approximately 10 m above the seabed, significantly higher than for the heads and bodies of the turbidity current, where the sediment typically reaches heights of approximately 5 m.

#### Quantitative analysis

**Fig. 7.**
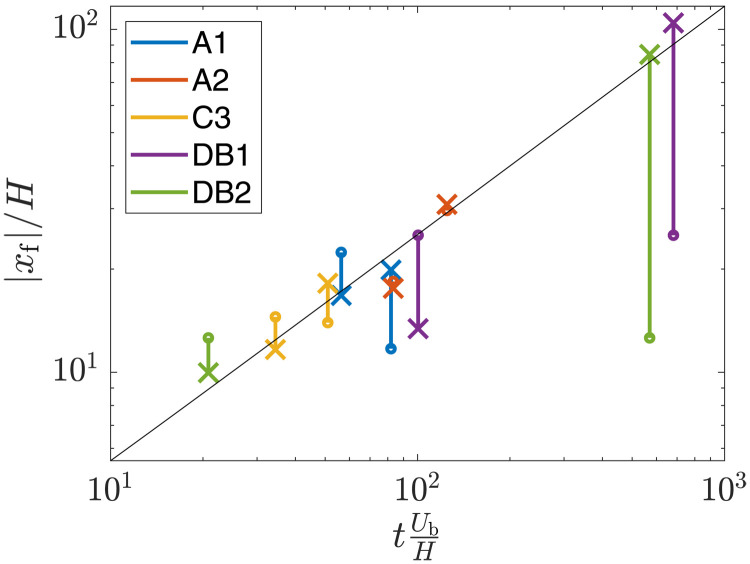
Turbidity current front positions measured during selfies and drive-bys. The crosses correspond to the front positions after removing the component of the background component in the direction normal to the tracks, providing an estimate of where the fronts would have been in the absence of a background flow. The front positions are shown in absolute value to show both fronts for each experiment. Note that both axes are logarithmic for readability. The black line is the front position predicted by the box model (see the “Box model” section in Methods).

To synthesize the findings on the role of crossflow on the front positions of the turbidity currents, Fig. 7 presents data on the two front positions for each of the three selfies for which background current data were available and for each of the two aforementioned drive-bys. The corresponding front positions in the absence of a crossflow component are then computed by subtracting the crossflow contribution, such that the corrected front positions are ${\hat{x}}_{f}(t)=x_{f}(t)-U_{r}\mathrm{pt}$. Following the findings of (*18*), we assume that *U*_⊥_ is 60% of the crossflow component, i.e., *U*_⊥_ = 0.6 *U*_cf_ (see Eq. 2, where, for a drive-by, **e**_**x**_ is defined as the unit vector normal to the track and defined positive toward the mooring). The corrected front positions, marked by the crosses in Fig. 7, are generally in much better agreement with the box model prediction than the measured front positions. This shows that the turbidity current generated by the collector can be assumed, in addition to spreading under the effect of its negative buoyancy, to interact hydrodynamically with the crossflow component of the background current, resulting in approximately 60% of the mean crossflow velocity acting to advect the center of mass of the turbidity current. The second front of C3 and the first front of DB1 are “overcorrected” under the above assumptions when compared with the box model. We also see that A2, for which the crossflow component is negligible and is thus mostly unaffected by the correction, agreement with the box model is not perfect. There are several other physical processes that could be playing a role here, such as potential asymmetries in the initial discharge, the slope of the local topography, intrinsic spatial and temporal heterogeneity of the turbidity current head, or the influence of the spanwise (i.e., along track) component of the current on the propagation of the turbidity current.

### Runout length

The time evolution of the turbidity current, data for which are presented in Fig. 4, suggests that the turbidity current is still propagating even at distances of ∼100 m away from the tracks, yet observations of the whole plume passing through the mooring during drive-by experiments (see the “Drive-by experiments” section in Methods) confirm that eventually the turbidity current has to propagate more slowly than the component of the background current that serves to advect the front. In the presence of a crossflow, the runout length of the resulting turbidity current is defined as the maximum distance reached by each of the fronts from the center of mass of the plume, which is being continuously advected by the background current. Consequently, in the presence of a crossflow, the runout length is different from the maximum distance reached by the turbidity current fronts with respect to the collector tracks. Box model approximations can be used to estimate the runout length of turbidity currents in the absence of a background crossflow, i.e., the maximum distance reached by the turbidity current before all particles settle down, reducing the negative buoyancy of the current to zero. For a monodisperse current, the box model runout length is given by (*22*)

$$L_{r}={\left(5\mathrm{FrA}\sqrt{g\prime A}/V\right)}^{2/5}$$ (3)

where *V* is the settling velocity of the particles, here assumed constant and independent of turbulent processes occurring in the current. Any level of polydispersity, however, increases the runout length when compared to a monodisperse current with the same weigh-averaged settling velocity (*22*). In addition, the equilibrium Eulerian description of particle settling that lies at the core of these simple models assumes that particles settle at a constant velocity within the flow field generated by the current and ignores particle-particle interaction as well as the interaction of turbulence and the stratification of the current that results from settling itself. The latter remains an unresolved question in turbidity currents (*23*), and many theories have been proposed to explain how turbulence and stratification interact to maintain particles in suspension for much longer time than anticipated, leading to so-called long-runout turbidity currents. Last, as the currents slow down over time, their velocity can become comparable to—and eventually smaller than—the background currents, at which point their dynamics might become primarily controlled by ambient hydrodynamic processes. Thus, predicting the maximum width of the deep seabed mining collector plume in the turbidity-current phase remains a challenge, even in full knowledge of the initial conditions and sediment properties of the discharge. Nevertheless, data from these field studies provide valuable insight into the scale of the collector plume width.

The data in Fig. 7 reveal that *U*_⊥_ ≈ 0.6 *U*_cf_ (see Eq. 2), in agreement with (*18*). The DB1 drive-by started at approximately 10:30, and the second front reached the mooring around 14:12, such that *t*_2_ ≈ 220 min. The collector had two collection pumps active, and the crossflow component of the background current was *U*_cf_ ≈ 4 cm/s. The DB2 drive-by started around 8:10, and the second front reached the mooring around 10:50, such that *t*_2_ ≈ 160 min. The collector drove with all four collection pumps active, and the crossflow component of the background current was *U*_cf_ ≈ 5 cm/s. Using the method derived in the “Estimating the runout length” section in Methods, we thus estimate that total distance between the fronts at time *t*_2_ was, assuming symmetry around the center of mass, *L* ≈ 440 m and *L* ≈ 476 m during DB1 and DB2, respectively. These estimated runout lengths are very similar despite the DB1 having been run with half the number of active collection heads, with DB2 reaching a distance between fronts 8.2% longer than DB1. The discharge mass flow rate was estimated to be approximately 9 and 12 kg/s for DB1 and DB2 (see the “Drive-by experiments” section in Methods), respectively, and note that in Eq. 3, the runout length scales with *g*^′2/10^ and thus with ${\overset{\cdot }{m}}^{2/10}$. Applying this scaling, we anticipate that the runout length of DB2 should be 7.8% longer than that of DB1, which agrees well with 8.2% increase in runout length calculated above. In the case of drive-by DB3, the plume was not observed at the mooring located 100 m north from the collector tracks because of the southward background current. However, the lack of detection does not imply that the runout length was less than 100 m because the background current was, at the same time, advecting the center of mass of the plume away from the mooring. With the available data, it is not possible to estimate the runout length of DB3; however, using the box model (see Methods) with a discharge of 9 kg/s (two collection pumps on) and assuming that 60% of the crossflow component (4 cm/s) is added to the front velocity, we find that the front propagating toward the mooring reaches a maximum distance of approximately 60 m from the tracks 1.5 hours after release. After this maximum is reached, the background current becomes the dominant advective mechanism, and the front position starts receding toward the tracks. This is consistent with the fact that no plume was observed at the mooring 100 m from the tracks during DB3.

Seabed slope is also a parameter known to influence the behavior and runout length of a turbidity current (*23*). For example, it has been observed that relatively weak slopes may influence spreading and even lead into autosuspension (*23*–*25*). In the area of the study, the slopes were small, in most cases below 1°, with two selfies and one drive-by conducted in areas with a slope between 1° and 1.5° (see Table 1 and fig. S1). We found no clear evidence of the influence of slope on the turbidity current spreading, which rather seemed to be primarily influenced by the background current. Although there was no clear influence from the slope in these experiments, the role of slopes and the existence of a critical angle for autosuspension are complex and active research topics (*23*), which may become important considerations in areas with steeper slopes on the abyssal plain.

Flocculation processes, including floc formation and floc breakage, could also potentially influence the spreading of the turbidity current, yet it is currently a poorly understood matter (*23*). Flocculation models have been proposed on the basis of both a posteriori turbidite observations [e.g., (*26*)] and laboratory experiments [e.g., (*4*, *27*)] but have not been applied or validated with in situ field data within a turbidity current. Both the concentration distribution and turbulence in turbidity currents are highly heterogeneous and transient, such that flocculation propensity observed in isotropic turbulence of homogeneous suspensions does not readily apply. In the selfie and drive-by experiments, the propagation distance of the turbidity current (Figs. 4 and 7) remains in good agreement with the box model prediction over the course of several tens of minutes despite settling being absent from the box model prediction (see the “Turbidity current modeling” section in Methods). Such good agreement suggests that a notable fraction of the sediment that became part of the turbidity current did not rapidly settle due to strong aggregation effects. This is further supported by the drive-by experiments, where suspended sediment is observed at greater heights above the seabed than during the earlier phase of the turbidity current observed during the selfies, indicating slow particle settling velocities and/or sufficient vertical transport by background turbulence. Thus, while flocculation could be present in the turbidity current, it does not markedly reduce the mass of suspended fines that most contribute to the runout length of a turbidity current (*22*). Flocculation processes may, however, play a more substantial role in the subsequent passive advection phase of the sediment by background currents. It might also be the case that for steeper bathymetry, with slopes of several degrees, a turbidity current could become self-sustaining and travel much longer distances, giving more potential for flocculation to play a role in the turbidity current dynamics.

### Sediment budget

The DB1 and DB2 drive-by datasets (see Fig. 6) provide some insight into the order of magnitude of the fraction of sediment that remains in suspension 2 m or more above the seabed after the collector has passed. For far-field indirect impact, a primary consideration is sediment that has, through some mechanism, been detrained either by turbulence directly behind the collector or from the gravity current. The moorings deployed for DB1 and DB2 saw the entire plume pass by as a result of the direction of the background current, and by assuming an advection velocity for the sediment, we can estimate the total mass per unit length of sediment that passed through the mooring 2 m or more above the seabed and compare that with the mass of sediment per unit length contained in the wake of the collector immediately after discharge. We focus on the second turbidity current that initially propagated in the direction opposed to the mooring, as it had more time to propagate and detrain before passing by the mooring. Thus, we only consider the data after the central patch, identified in Fig. 6, has passed by the mooring.

Assuming that the sediment is passively advected by some fraction *k* of the crossflow component of the background velocity, the mass per unit length of sediment that passes through the mooring during the DB1 and DB2 drive-bys can be estimated as $m\approx kU_{c.f.}\int_{t_{c}}^{t_{2}}\int_{z_{1}}^{z_{2}}c\text{dzdt}$, where *t*_c_ and *t*_2_ are the times at which the central patch and the second front pass through the mooring, respectively, and *z*_1_ and *z*_2_ are the vertical positions of the lowest and highest STMs mounted on the collector, respectively. Following the findings in the “Role of background currents” section, we take *k* to be between 0.6 and 1, as it is not known whether the detrained sediment is uniformly advected by the whole crossflow component or a fraction of it.

In the case of DB1, the component of the background current that is normal to the track is *U*_cf_ ≈ 4 cm/s. Integrating the data of Fig. 6, we find that the mass per unit length *m* of suspended sediment that passed through the mooring between the lowest and highest STM (located 2 and 8.2 m above the seabed, respectively) can be estimated to be between 0.1 and 0.17 kg/m. Here, *m* can be compared to the initial mass per unit length contained in a slice of the wake of the collector immediately after discharge, which we found to be well approximated by $\frac{\overset{\cdot }{m}}{U_{c}}$, with $\overset{\cdot }{m}$ as the discharge in kilogram per second and *U*_c_ as the collector speed in meter per second. In the case of DB1, we find $\frac{\overset{\cdot }{m}}{U_{c}}\approx 18$ kg/m, such that the total mass per unit length *m* of sediment that passed through the mooring after the central patch during DB1 is approximately 0.5 to 1% of the initial discharge. If we assume that the turbidity current that was released in the direction of the mooring and that therefore passed through the mooring before detrainment could occur (see again Fig. 6) will produce a similar amount of detrainment, then we can anticipate that the total sediment per unit length produced by detrainment during DB1 is ∼2 kg/m, i.e., 1 to 2% of the initial discharge. Applying the same process to DB2, we find that *m* is between 0.54 and 0.9 kg/m, while $\frac{\overset{\cdot }{m}}{U_{c}}\approx 25$ kg/m. Thus, the total mass per unit length *m* of sediment that passed through the mooring 2 m or more above the seabed after the central patch during DB2 is approximately 2 to 4% of the initial discharge, and again, assuming similar conditions for the other side of the discharge, the total sediment per unit length produced by detrainment during DB2 is between 4 and 8% of the initial discharge. Here, we remind the reader that the sediment concentration is only known above a height of approximately 2 m. It is possible that more sediment can detrain or be transported by some turbulent mechanism from below the window of observation, resulting, at later times, in higher detrained fractions than calculated above.

## DISCUSSION

Our field experiments show that, as a result of the negative buoyancy induced by particle loading, the discharged sediment from a preprototype collector vehicle propagates as a turbidity current immediately after release. The turbidity current deposits sediment as it propagates and interacts with background currents through complex processes that affect the position of the turbidity current fronts and also the shape and vertical distribution of sediment concentration in the turbidity current. The studies were conducted in mostly flat areas (see Table 1 and fig. S1), and there was no clear evidence of the influence of slopes, although steeper seabed slopes could be a factor for the spreading of such a turbidity current in other parts of the abyssal plain. All qualitative and quantitative analyses of the data were consistently in good agreement with existing understanding of turbidity currents. This provides a clear physical picture of what the collector plume looks like behind a collector vehicle, affecting assumptions about initial conditions for modeling efforts, which have hitherto been necessarily ad hoc and lacking sufficient physical insight.

Of key environmental interest is the ratio of the amount of sediment deposited locally behind a collector vehicle compared to the amount of sediment that is detrained and remains in suspension. The two principal sources of detrained sediment are (i) that which is suspended due to direct interaction with the turbulent wake behind the vehicle (the central patch) and (ii) that which is detrained from the turbidity current as it propagates laterally away from the collector tracks. The observations suggest that 92 to 98% of the sediment mobilized by the collector were below 2 m at the time and location of the observations, with some local sediment deposition causing blanketing of nearby nodule fields (see fig. S6), while 2 to 8% of the sediment were 2 m or more above the seabed. Over a longer time scale, vertical turbulent diffusion near the seabed is the mechanism by which some of the sediment in suspension below 2 m could still be raised further above the seabed, in which case the amount of sediment dispersed away from the mining track could exceed the aforementioned 2 to 8%. This sediment budget is something that has yet to be properly accounted for in efforts to model collector plumes. Further studies focused on the detrainment of sediment from the turbidity current phase of the collector plume are needed to build upon these results and better inform the initial conditions of far-field ambient plume models.

During the 3 hours of plume evolution captured during the DB1 and DB2 experiments (see the “Drive-by experiments” section in Methods), the sediment was not only observed to stay in suspension but also to be transported vertically, resulting in detectable sediment concentrations above 3 m after several hours of evolution compared to typical selfie maneuvers, during which sediment rarely reaches above 3 m. This upward vertical transport of sediment during the turbidity current phase even at late times in its evolution shows that once the sediment detrains, it seems to remain in suspension, likely by a combination of factors, such as residual turbulence from the turbidity current, ambient shear-induced turbulence caused by background currents, or high degrees of sediment polydispersity resulting in a large fraction of sediment having a settling velocity orders of magnitude smaller than the mean. This has fundamental implications for the modeling of the ambient plume that results from detrainment. The Rouse number, defined as $P=\frac{V}{\kappa u*}$—where *V* is the particle settling velocity, κ ≈ 0.41 is the von Kármán constant, and u* is the friction velocity, typically around 5 to 10% of the mean flow—is often used to characterize the ability of turbulence to maintain sediment in suspension despite its ability to settle in a quiescent environment (*23*). It has been argued (*28*) that sediment remains in suspension for values of *P* < 1. Taking u^*^ as 5% of a characteristic background current of velocity 5 cm/s and assuming a mean individual settling velocity of 0.1 mm/s, the typical of sediment from the CCZ (*4*, *29*), we find that *P* is of order 0.1. The nonlinear nature of particle-particle and particle-fluid interactions is complex and cannot be reduced to the Rouse number (*23*), but the drive-by observations, combined with this simple dimensional analysis, confirms that the seabed plume, at least in the turbidity current phase, cannot be assumed to settle at the Stokes settling velocity of individual particles.

The cohesivity of the seabed sediment in this region of the ocean (*4*) makes flocculation processes, including floc formation and floc breakage, likely to occur within the turbidity current. Given the complexity of turbulent processes within turbidity currents (*23*), the role of flocculation on their evolution is poorly understood. The sediment concentrations observed in the head of the turbidity current were *O*(100) mg/liter and subject to varying levels of shear, which are conducive to flocculation (*4*). In our studies, however, the propagation distance of the turbidity current (Fig. 4) remains in good agreement with box model predictions over the course of several tens of minutes despite settling being absent from the box model prediction, suggesting that a notable fraction of the sediment that became part of the turbidity current did not rapidly settle due to strong flocculation effects. Rather, the sediment played a relatively passive role of influencing buoyancy in the turbidity current. Thus, while flocculation processes are very likely present in the head of the turbidity current, it does not seem to markedly reduce the mass of suspended fines, which contribute the most to the runout length of the turbidity current (*22*). Behind the head of the turbidity current, where concentrations of sediment left in suspension by the passing gravity current head were *O*(1) mg/liter, further flocculation is unlikely to occur due to the low concentration (*4*). On the other hand, any flocculation that did occur during the turbidity current phase is likely to influence the settling properties of the sediment left in suspension and subsequently advected by the background currents. Our drive-by experiments, which observed suspended sediment several hours later at higher heights above the seabed than during the earlier selfie phase of the turbidity current suggest some combination of slow floc settling velocities and vertical transport by background turbulence.

Last, it is worth recalling that the existence of the turbidity current regime, demonstrated here for the GSR collector under nominal operation parameters, depends fundamentally on the balance of forces that control the immediate vicinity of the discharge. As a thought experiment, let us consider a collector that discharges sufficiently little sediment in its wake that the negative buoyancy imparted by particle-loading is substantially weaker than the turbulence forces of the wake or than the background current magnitude and turbulence intensity. In such a scenario, the collector plume would skip the turbidity current phase and immediately enter the so-called ambient plume phase, where sediment transport is on the first order controlled by advection by background current, turbulent diffusion, and settling. On the one hand, this collector design discharged a much smaller amount of sediment than the collector that generated a turbidity current. On the other hand, all of the sediment was made readily available for far-field transport by background currents, unlike the collector that produces a turbidity current, which maintains the bulk of the sediment-laden fluid very close to the seabed and increases the prospects for local deposition [see fig. S6 and, for instance, (*14*)]. For any collector design, this further stresses the necessity of a thorough assessment of the balance of forces, operational parameters, expected plume regime, and fraction of discharged sediment made available for far-field transport as a result of both wake mixing processes and detrainment from the turbidity current. In particular, a wide range of collector designs and sizes with different operational parameters might be considered. The balance of forces in the wake does not vary linearly with operational parameters, and the operational parameters themselves might not vary linearly with scale, which could result in fundamentally different operational regimes.

## METHODS

### Selfie experiments

#### Maneuvers

**Fig. 8.**
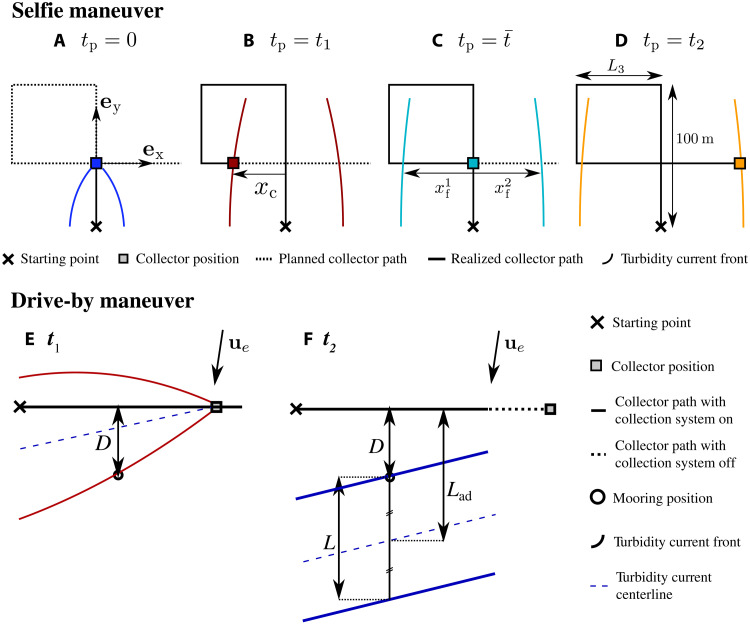
Sketch of the selfie and drive-by maneuvers. The selfie maneuver (**A** to **D**) illustrates the collector position (colored square), turbidity current fronts positions (colored lines), the realized color path, and planned collector path (full and dashed black line, respectively), at four different times. The propagation time *t*_p_ is set to start when the collector passes the intersection point (A). The maneuver is illustrated at time (A) *t*_p_ = 0, (B) *t*_p_ = *t*_1_, when the collector encounters the first turbidity current front, (C) $t_{p}=\bar{t}$, when the collector crosses over its own tracks, and (D) *t*_p_ = *t*_2_, when the collector exits the second turbidity current front. *x*_c_ denotes the position of the collector, while $x_{f}^{i}$ denotes the position of the turbidity current front. In the case of the drive-by maneuver, (**E**) the first front reaches the mooring at time *t*_1_, with its propagation direction aligned with the direction of the crossflow component of the background current. (**F**) The second front reaches the mooring at time *t*_2_ as a result of interaction with, and advection by the crossflow component of the background current. Provided that the intrinsic front velocity has considerably decreased by time *t*_2_, the distance between the fronts *L* can be estimated as *L* ≈ 2(*L*_ad_ − *D*), where *L*_ad_ is the estimated distance between the track and the center of mass of the turbidity current and *D* is the distance between the track and the mooring.

The goal of the selfie maneuver is to produce and monitor a disturbance by collecting nodules driving in a straight line, which will be the most standard component of a normal mining operation, and intersecting the resulting sediment plume with the collector vehicle, thereby measuring the properties of the plume with instrumentation mounted on the collector itself. During each selfie maneuver, the collector first drove a ∼100-m track (*L*_1_) collecting nodules before turning off its collection pumps, conducting three 90^∘^ turns, and proceeding to drive perpendicularly across this track (*L*_4_), thereby encountering its own plume (see Fig. 8).

While we anticipated that the collector would outpace the turbidity current generated during the nodule collection section of the maneuver, considerable uncertainty remained as to how quickly the current would propagate, and thus when in the course of the maneuver, the collector would encounter its own plume. A conservative approach was therefore adopted using a simple box model [see (*22*)] to predict the propagation of the current in the absence of settling and deposition and designing a reference selfie maneuver that guarantees that the plume will not be encountered until the last segment of the maneuver. As the experiment progressed and a better understanding of the gravity current propagation was obtained, the segments of the maneuver were iteratively shortened to intersect the plume earlier and earlier in its propagation.

A sketch of snapshots of the selfie maneuvers is shown in Fig. 8 to illustrate the relative position of the collector and turbidity current front at various times. Under the assumption that the current propagates mainly in the direction normal to the track [see (*10*)], a propagation time *t*_p_ is defined as the difference between the measurement time and the turbidity current release time. This release time is taken as the time at which the collector passed through the point where the path intersects itself (Fig. 8A). The maneuvers are designed such that the collector encounters the first current front only during the last segment of the selfie, at time *t*_1_ (Fig. 8B). The time at which the collector crosses over its track is identified as time $\bar{t}$ (Fig. 8C). Last, given that the front velocity of the current is smaller than that of the collector and that the former decreases with time, the collector is guaranteed to exit the plume through the second front, at time *t*_2_ (Fig. 8D). In the coordinate system of the selfie, the position of the collector along this *x* axis is denoted as *x*_c_ (Fig. 8B), while the position of the left and right propagating current fronts on the *x* axis are denoted $x_{f}^{1}$ and $x_{f}^{2}$ respectively (Fig. 8C). By definition, *t*_1_ and *t*_2_ are such that $x_{c}(t_{1})=x_{f}^{1}(t_{1})$ and $x_{c}(t_{2})=x_{f}^{2}(t_{2})$ and thus depend on the temporal evolution of the fronts. Given the uncertainty associated with predicting the velocity of the front, we cannot accurately control *t*_1_ and *t*_2_. Instead, we progressively reduce the distance *L*_3_ (Fig. 8D) in each successive maneuver, thereby reducing the distance covered by the collector before it enters and exits the current, doing so at earlier and earlier times.

A total of eight selfie experiments were conducted during the field studies (Table 1). The slope in the areas where the selfie experiments was below 1° for most selfies, with two of them conducted in areas with a slope between 1° and 1.5°. For six of the experiments, all the collection system parameters were kept constant, and only the length of segment *L*_3_ (see Fig. 8) was modified to intersect the turbidity current at different times. For the first two selfie experiments, *L*_3_ was set to a conservative value of 130 and 170 m, respectively, and it was progressively decreased down to 60 m. For experiment A1, two of the four collection pumps were pumping about 50% of the nominal flow rate. For experiment B1, the collection system was turned off, and the measured sediment plume was only created by the tracks of the collector vehicle. A top view of all the selfies is presented in fig. S2 that shows the sediment concentration measured by the lowest mounted STM on the collector vehicle along the course of the maneuver.

#### Selfie instrumentation

**Fig. 9.**
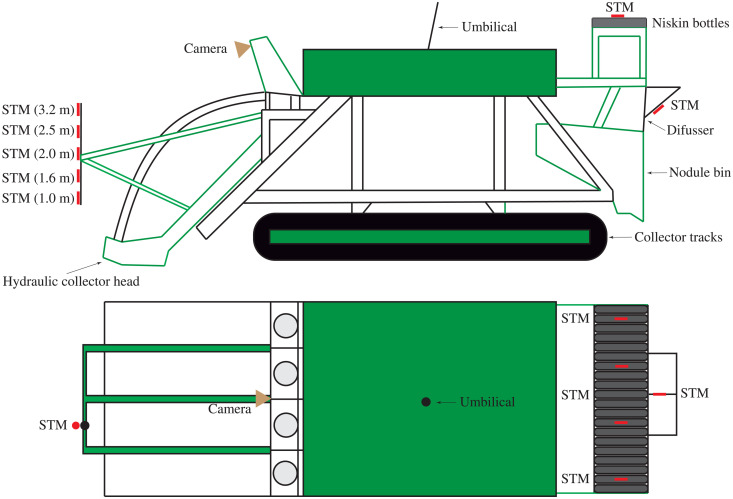
Schematic of the instrumentation mounted on the collector vehicle. The instrumentation setup included a total of 10 STM turbidity sensors and a forward-looking video camera.

The nodule collector vehicle was equipped with substantial monitoring equipment at the front (as it can be seen in Fig. 5) and rear to conduct the selfies (Fig. 9). A total of 10 optic STMs were mounted on the vehicle, 5 at the front on a pole reaching from 1 to 3 m above the ground, 4 across the top of the vehicle to accompany a set of 20 2.5 l Niskin sample bottles, and 1 at the rear in the vicinity of the discharge vents. Internal instrumentation monitored the flow rate and sediment concentration within the vehicle.

Nine of the 10 STMs on the collector vehicle (Fig. 9) were directly powered by the collector vehicle and sent data in real time back to the vessel. The other STM was mounted on a self-logging and self-powered Aquatec AQUAlogger 310YT unit. All the STMs were sampled at 1 Hz and were calibrated beforehand in the laboratory using seabed sediment from the field studies area, as detailed in the “Seapoint Turbidity Meter calibration” section in the Supplementary Materials. The STMs ambient signal was removed to only consider the actual plume sediment concentration.

A set of internal pressure sensors (Keller Series 35X) and one sediment concentration sensor (MIX-ITOMETER) were mounted inside the ducts of the nodule collection system to estimate the water flow rate and measure the sediment concentration before the discharge. The data from the instrumentation were applied to a simple discharge model to estimate the sediment mass flux (*m*), which is the parameter of interest to set the initial conditions of the turbidity current. A MIX-ITOMETER sediment concentration sensor developed by Industrial Tomography Systems (ITS) was mounted in the sediment discharge duct to measure the sediment concentration of the discharge. The sensor consists of 18 electrodes mounted evenly spaced on a rod to measure the sediment-laden fluid electrical properties. The MIX-ITOMETER was cross-calibrated using a previously calibrated STM and seabed sediment obtained from the area where the field studies were taking place. The calibration results showed a very linear behavior within the sediment concentration range of interest (see the “MIX-ITOMETER calibration” section in the Supplementary Materials).

#### Sediment output model

The data from the pressure sensors were translated into flow velocity based on a previous calibration in shallow water conducted by the manufacturer of the collector vehicle. The flow velocity was then multiplied by the area of the ducts to obtain the volume flux, which multiplied by the sediment concentration measured by the MIX-ITOMETER provides the discharged sediment mass flux. On the basis of previous studies conducted by the manufacturer of the collector vehicle, the collection system entrains as much water from the background ocean at the front as the pumping system injects.

### Drive-by experiments

#### Maneuvers

In a drive-by experiment, a disturbance is produced by the collector vehicle driving a straight track passing at a minimum distance *D* from an instrumented mooring (see Fig. 8, E to F). A total of four drive-by experiments were conducted during the field studies, but only two of them (DB1 and DB2) resulted in clear plume signals because of a combination of the distance between the collector track and the mooring and the background ocean current velocity and heading (Table 1). The plume released during drive-by DB3 did not reach the mooring because of the opposing background current. The fourth drive-by experiment (DB4) was conducted with the collector vehicle driving 150 m northward with the collection system active, 100 m away to the West from the instrumented mooring (see fig. S1), and with a 7-cm/s southward background current (see table S1). A signal arrived to the mooring 1 hour later, which indicates that it was initiated further north after the end of DB4 and while the collector vehicle was conducting other sampling activities that were not part of this study and so not well constrained.

#### Mooring instrumentation

Mooring MA (fig. S4A) was deployed during selfie experiments A1 and A2. The mooring had a total of six STMs at heights between 2.5 and 8.2 m above the seabed to measure sediment concentration. Four of the STMs were connected to an RBRduo logging and powering unit sampling every 3 s, and the two other STMs were connected to a Seabird CTD SBE 16plus sampling every 10 s. The STM’s ambient signal was removed to produce Fig. 6 and fig. S5 and to determine the sediment budget so that only the plume sediment concentration is used (see the STM calibration section in the Supplementary Materials). Two ADCPs were mounted back to back on a buoy ∼17 m above the seabed to measure ocean currents and detect the acousticbackscatter signal of the sediment plume. A 300-kHz Teledyne Workhorse ADCP was mounted looking up, and a 600-kHz Teledyne Workhorse was mounted looking down. Both instruments were set up to sample with a resolution of 1 m and a frequency of 1 Hz. Additional instrumentation was mounted on the mooring for other purposes, such as transponders for positioning and thermistors for turbulence measurements.

Mooring MC (fig. S4B) was deployed during selfie experiment C3. In this case, the mooring had three STMs at 1.9, 2.6, and 4.6 m above the seabed; two of them were powered by an RBRduo logger sampling every 3 s, and the third one was mounted on an Aquatec AQUAlogger unit sampling every second. The same two ADCPs used in mooring MA were also mounted on mooring MC ∼17 m above the seabed.

### Turbidity current modeling

#### Discharge characterization

While picking up nodules, the GSR collector discharged, on average, 12 ± 3 kg/s of sediment ($\overset{\cdot }{m}$) in its wake and moved at an average speed of *U*_c_ ≈ 0.3 m/s. Assuming that the sediment discharge is quickly mixed over the collector wake area, which we take to be equal to the collector’s cross-sectional area (*10*), then the sediment concentration in the wake is approximately $c_{0}=\frac{\overset{\cdot }{m}}{U_{c}\mathrm{WH}}$, with *H* and *W* the collector’s height and width, respectively. Following (*22*), a reference buoyancy velocity can be defined as $U_{b}^{0}={\left(g\prime \sqrt{A}\right)}^{\frac{1}{2}}$, where $g\prime =g\frac{\overset{\cdot }{m}}{U_{c}H^{2}}\frac{\rho_{p}-\rho_{w}}{\rho_{w}\rho_{p}}$ is the reduced gravity due to particle loading in the wake, *g* is gravitational acceleration, ρ_p_ ≈ 2600 kg/m^3^ is the density of the particles, ρ_w_ ≈ 1030 kg/m^3^ is the density of ambient water. In (*22*), *A* is the area of the dense fluid being released in the rectilinear lock-release configuration. By symmetry, we assume that half of the sediment discharged behind the collector will propagate to the left side of the track, while the other half will propagate to the right. As a result, we take *A* to be half the area of the wake, i.e., $A=\frac{HW}{2}$. Given that *H* = *W* ≈ 4 m, the buoyancy velocity is therefore *U*_b_ ≈ 0.21 m/s for a typical selfie. This suggests that the sediment plume discharged by the collector will form a fast-propagating gravity current that moves at a velocity comparable to—yet smaller than—the speed of the collector itself. Following (*10*), we further anticipate that it will propagate mainly in the direction normal to the tracks.

Certain experiments were run with only two collection pumps active or with no collection pumps active. When the pumps are not running, as it was the case during selfie B1, the top layer of sediment usually removed by the pumping mechanism remains unperturbed but can then be resuspended by the collector tracks themselves. This resuspension mechanism is poorly understood, and the discharged mass flux is not well characterized in this case. As a result, the mass flux during B1 was determined by fitting the results of Fig. 4 to the box model approximation, and we found that approximately 3 ± 2 kg/s of sediment. is picked up by the tracks when all pump heads are turned off. When half of the four collection head pumps are active, a discharge of 9 ± 2 kg/s is assumed, which combines half of the track pickup and half of the pumps of the two extremes.

#### Box model

Given that the collector velocity is larger than the buoyancy velocity estimated in the “Discharge characterization” section in Methods and following observations that the effective front velocity of the turbidity current is smaller than the buoyancy velocity, we model the turbidity current propagation using a simple lock-release box model of a turbidity current propagating in the direction normal to the direction of motion of the collector (*10*). We use the box model of (*22*), and assuming that the sediment in the wake will initially form two symmetric currents on each side of the collector, we consider an initial lock of height *H* and length *H*/2, where *H* is the height of the collector. There is considerable uncertainty on the effective settling velocity distribution of the suspended sediment, which may greatly differ from the settling velocity distribution in a quiescent fluid owing to the complex turbulent processes taking place within the turbidity current (*23*). The box model is therefore considered in the absence of any settling.

#### Estimating the distance between the two fronts

Following (*18*), the front positions $x_{f}^{1}$ and $x_{f}^{2}$ are assumed to be equal to the sum of the contribution from the turbidity current front position *x*_*t*.*c*._ in the absence of a crossflow and of the contribution from the crossflow component of the background current, that is, $x_{f}^{1}=-x_{t.c.}(t)+U_{⊥}t$ and $x_{f}^{2}=x_{t.c.}(t)+U_{⊥}t$, where *x*_*t*.*c*._ is the front position of the gravity current in the absence of crossflow, and *U*_⊥_ is the component of the background current normal to the track that advects the current, and the signs reflect the direction of propagation of fronts 1 and 2. We can then estimate the error made by approximating the distance between the fronts *L*_f_ in Eq. 1 through Taylor series expansion of the front position $x_{f}^{1}$ at time *t*_1_ and $x_{f}^{2}$ at time *t*_2_ around the mean time $\frac{t_{1}+t_{2}}{2}$. We find that

$${x_{f}^{1}(t_{1})=-x_{g.c.}(\frac{t_{1}+t_{2}}{2})+\frac{\delta t}{2}\frac{\partial x_{g.c.}}{\partial t}|}_{\frac{t_{1}+t_{2}}{2}}-\frac{\delta t}{2}U_{⊥}+O(\delta t^{2})$$ (4)

$${x_{f}^{2}(t_{2})=x_{g.c.}(\frac{t_{1}+t_{2}}{2})+\frac{\delta t}{2}\frac{\partial x_{g.c.}}{\partial t}|}_{\frac{t_{1}+t_{2}}{2}}+\frac{\delta t}{2}U_{⊥}+O(\delta t^{2})$$ (5)

where δ*t* = *t*_2_ − *t*_1_ and the *O*(δt^2^) term is proportional to the rate of change of the front velocity. Thus, with *L*_f_ = 2*x*_*t*.*c*._, we find

$$x_{f}^{2}(t_{2})-x_{f}^{1}(t_{1})=L_{f}(\frac{t_{1}+t_{2}}{2})+\delta tU_{⊥}+O(\delta t^{2})$$ (6)

In general, the background currents during the selfie experiments did not exceed 6 cm/s, and, as further discussed in the “Role of background currents” section, only approximately 60% of the component of the background current that is aligned with the direction of propagation of the turbidity current acts to translate the front positions of the turbidity current [see also (*18*)].

#### Estimating the runout length

During drive-bys DB1 and DB2, the first patch of sediment encountered at the mooring consists of the front propagating in the direction of the crossflow (Fig. 8A), while the last patch of sediment observed at the mooring (Fig. 8B) consists of the front propagating against the crossflow, which has slowed sufficiently (or stopped) such that there is a net advection velocity in the direction of the background flow, toward the mooring. It is not known whether the turbidity current has reached its runout length at the time the last front is encountered. Nevertheless, we can estimate some bounds for the runout length by considering the propagation of the second front relative to the predicted position of the center of mass of the current. We can simply assume that the center of mass of the turbidity current at time *t*_2_, when the second front passes through the mooring, is located at a distance of *L*_ad_ = *U*_⊥_*t*_2_ from the tracks (Fig. 8B). The total spread of the current is thus, by symmetry, equal to twice the distance between the center of mass and the second front, which is by definition located at the mooring, 50 and 100 m from the tracks during DB1 and DB2, respectively. That is, the total spread, sketched in Fig. 8B is given at time *t*_2_ as

$$L\approx 2(L_{\text{ad}}-x_{f}^{2}(t_{2}))=2(L_{\text{ad}}-D)$$ (7)

where we recall that *D* is the distance between the track and the mooring.

## Supplementary Materials

### This PDF file includes:

Selfie and drive-by experiments map Selfie experiments top view Background currents Moorings Drive-by ADCP backscatter intensity Local deposition observed 14 hours after selfie C2 Seapoint Turbidity Meters calibration MIX-ITOMETER calibration Table S1 Figs. S1 to S8 References

## Notes

View/request a protocol for this paper from *Bio-protocol*.
