# Supplementary material for: An in situ study of abyssal turbidity-current sediment plumes generated by a deep seabed polymetallic nodule mining preprototype collector vehicle
Source: Sci Adv. 2022 Sep 21;8(38):eabn1219. doi: 10.1126/sciadv.abn1219 (PMC9491711; doi:10.1126/sciadv.abn1219)
Supplement: Supplementary file 1 — Selfie and drive-by experiments map Selfie experiments top view Background currents Moorings Drive-by ADCP backscatter intensity Local deposition observed 14 hours after selfie C2 Seapoint Turbidity Meters calibration MIX-ITOMETER calibration Table S1 Figs. S1 to S8 References [file sciadv.abn1219_sm.pdf]

Science Advances
AAAS

Supplementary Materials for

An in situ study of abyssal turbidity-current sediment plumes generated by a deep seabed polymetallic nodule mining preprototype collector vehicle

Carlos Muñoz-Royo *et al.*

Corresponding author: Carlos Muñoz-Royo, carlosmr@mit.edu; Thomas Peacock, tomp@mit.edu

Sci. Adv. **8**, eabn1219 (2022)
DOI: 10.1126/sciadv.abn1219

This PDF file includes:

Selfie and drive-by experiments map
Selfie experiments top view
Background currents
Moorings
Drive-by ADCP backscatter intensity
Local deposition observed 14 hours after selfie C2
Seapoint Turbidity Meters calibration
MIX-ITOMETER calibration
Table S1
Figs. S1 to S8
References

Selfie and drive-by experiments map

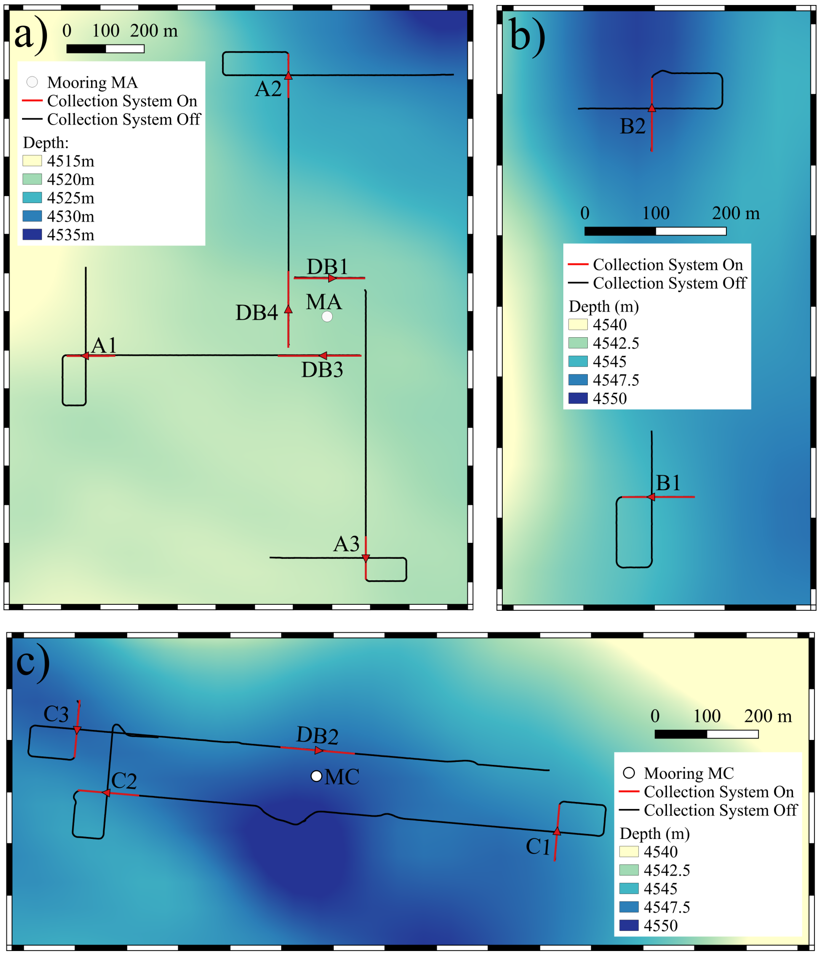

Figure S1: Topographic map of the A (a), B (b), and C (c) experiment sites. The red and black lines correspond to the tracks of the collector vehicle with the collection system turned on and off, respectively. The arrows indicate the driving direction of the collector vehicle. The selfie and drive-by experiments are labeled (see Table 1). Note that both the scale and color map for each location are different for better visualization. The bathymetry data was obtained from (*19*).

Selfie experiments top view

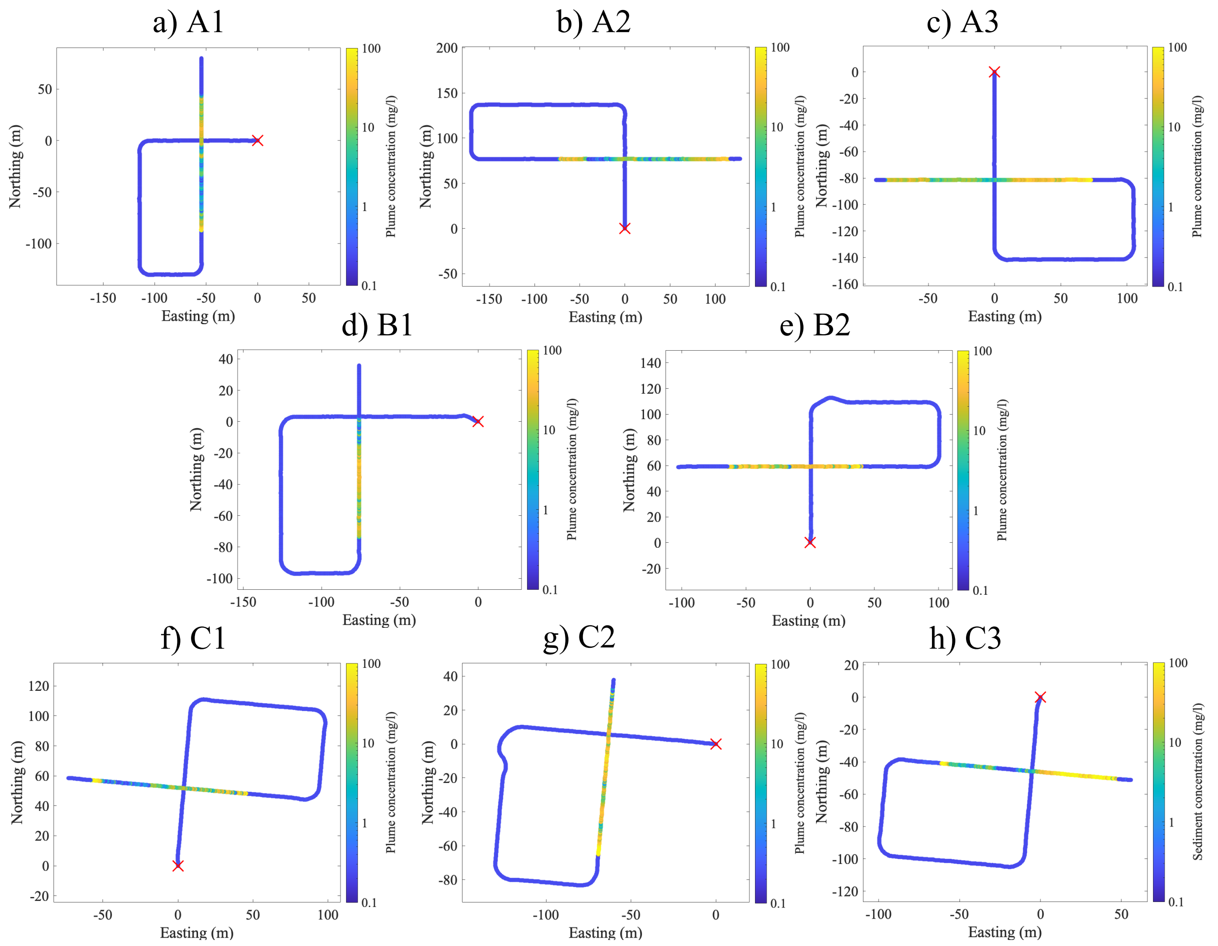

Figure S2: Top view of all the selfie experiment in the Easting (x) - Northing (y) coordinate system centered on the starting position of each selfie. The color of the tracks corresponds to the plume sediment concentration above ambient levels measured by the lowest collector-mounted STM at that particular position of the maneuver. The lowest and highest concentrations in the color map do not necessarily correspond to the ocean background concentration or the highest measured concentration, respectively.

Background currents

The downward looking 600kHz Teledyne Workhorse ADCP (mounted 16m above the seabed on moorings MA and MC) was able to measure the ocean current velocity and heading between 7m and 10m above the seabed (Figure S3). The ADCP was sampling at 1Hz in beam coordinates with a bin size of 0.5m, and the data was smoothed applying a 20-minute moving average. It was not possible to obtain current data of good quality below 7m because of the side lobe reflections of the acoustic beams on some of the instrumentation mounted closer to the seabed. Mean current velocities were between 4cm/s and 7cm/s during the selfie and drive-by experiments in which the moorings were deployed, heading mostly to the South (Table S1). The background velocities used to produce Figure 7 were calculated by averaging the ADCP data between 7m and 10m above the seabed.

Table S1: Mean current velocity eastern (u) and northern (v) components measured between 7m and 10m above the seabed during four of the experiments.The time interval considered to calculate the mean is listed in the fifth column. In the case of the drive-bys DB1 and DB2, the time interval corresponds to the time between the start of the drive-by and the last plume observation at the mooring. In the case of DB3 and DB4, in which there was no direct sediment plume observation, the interval is four hours. In the case of the selfies, the time interval starts with the start of the selfie and finishes at the time the collector vehicle exited the plume in the last leg of the selfie maneuver.

Experiment	u (m/s)	v (m/s)	Mooring	Time interval (min)
DB1	0.00	-0.04	MA	245
DB2	-0.03	-0.05	MC	173
DB3	0.00	-0.04	MA	240
DB4	-0.01	-0.07	MA	240
A1	0.00	-0.04	MA	30
A2	-0.01	-0.07	MA	38
C3	-0.04	-0.05	MC	17

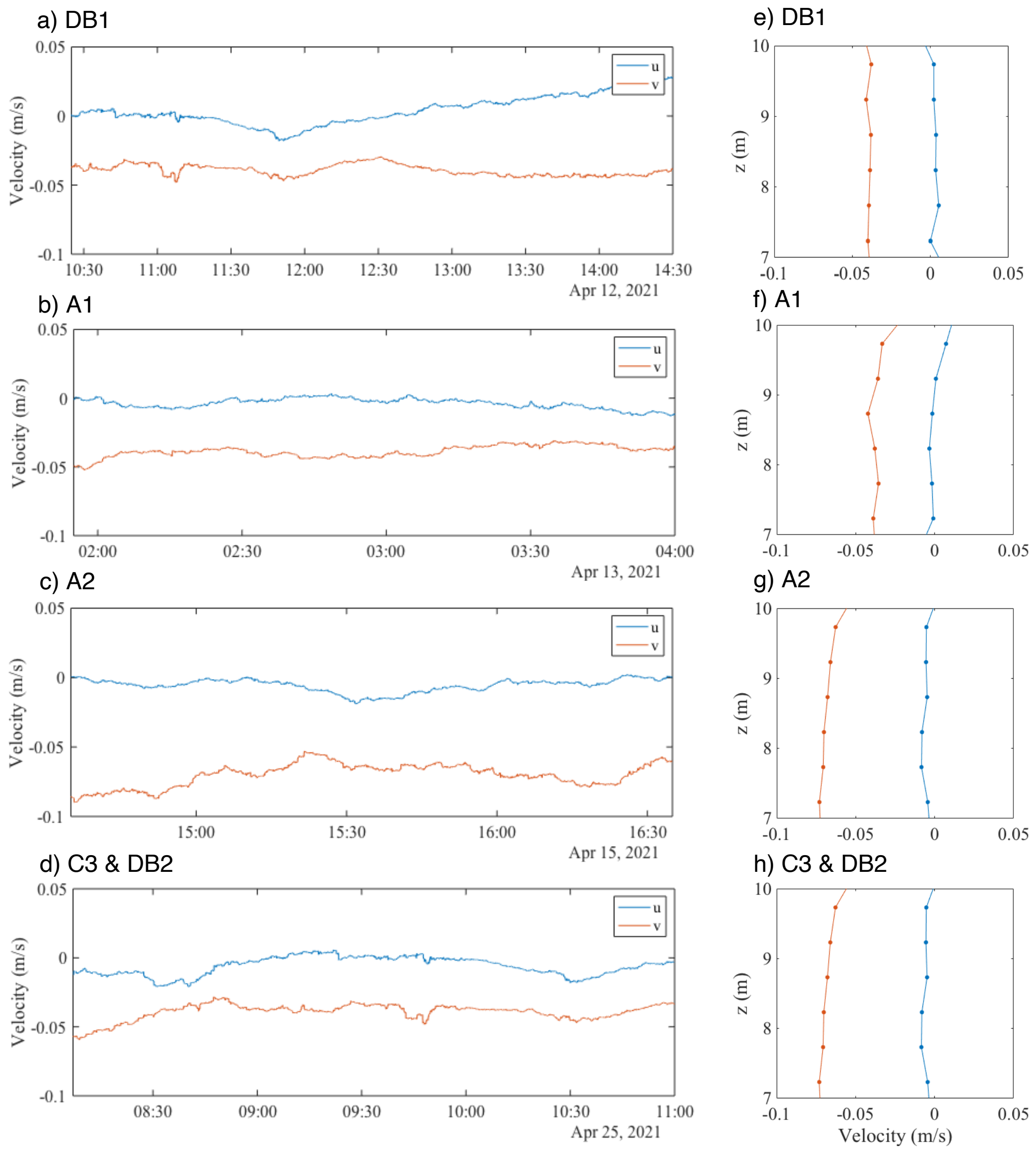

Figure S3: Time series and mean vertical profile of the current velocity measured by the downward looking 600kHz ADCP on moorings MA and MC. The time series show the 30-minute moving average of the mean current velocity components between 7m and 10m above the seabed before, during and after each experiment. Mooring MA was deployed for selfie and drive-by experiments A1, A2, and DB1. Mooring MC was deployed for C3 and DB2 experiments. The blue and red lines correspond to the East (u) and North (v) component of the measured current velocity, respectively.

Moorings

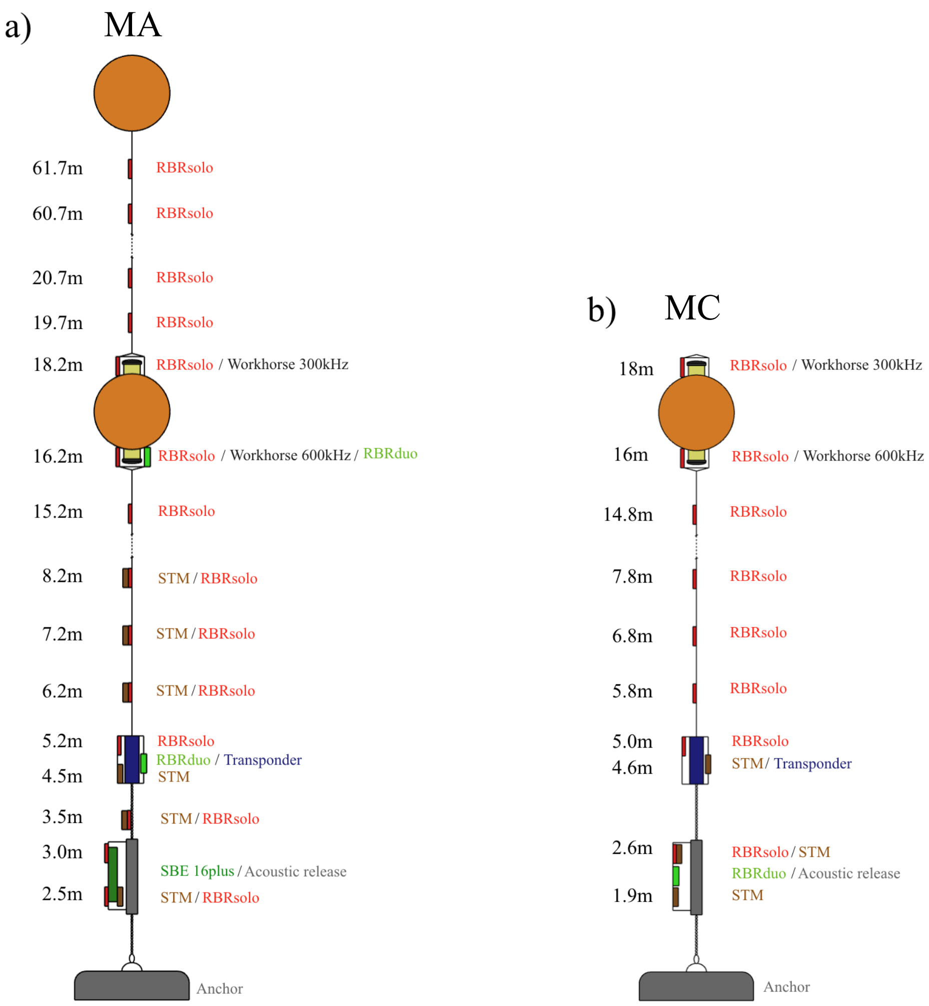

Figure S4: Schematic of moorings (a) MA and (b) MC. For the purpose of this study, the moorings were equipped with STMs (in brown) to measure sediment concentration at different heights, and two ADCPs to measure the ocean currents close to the seabed. In both moorings, the upward looking ADCP is a 300kHz Teledyne Workhorse, while the downward looking is a 600kHz Teledyne Workhorse. A number of RBRsolo thermistors were also mounted on both moorings.

Drive-by ADCP backscatter intensity

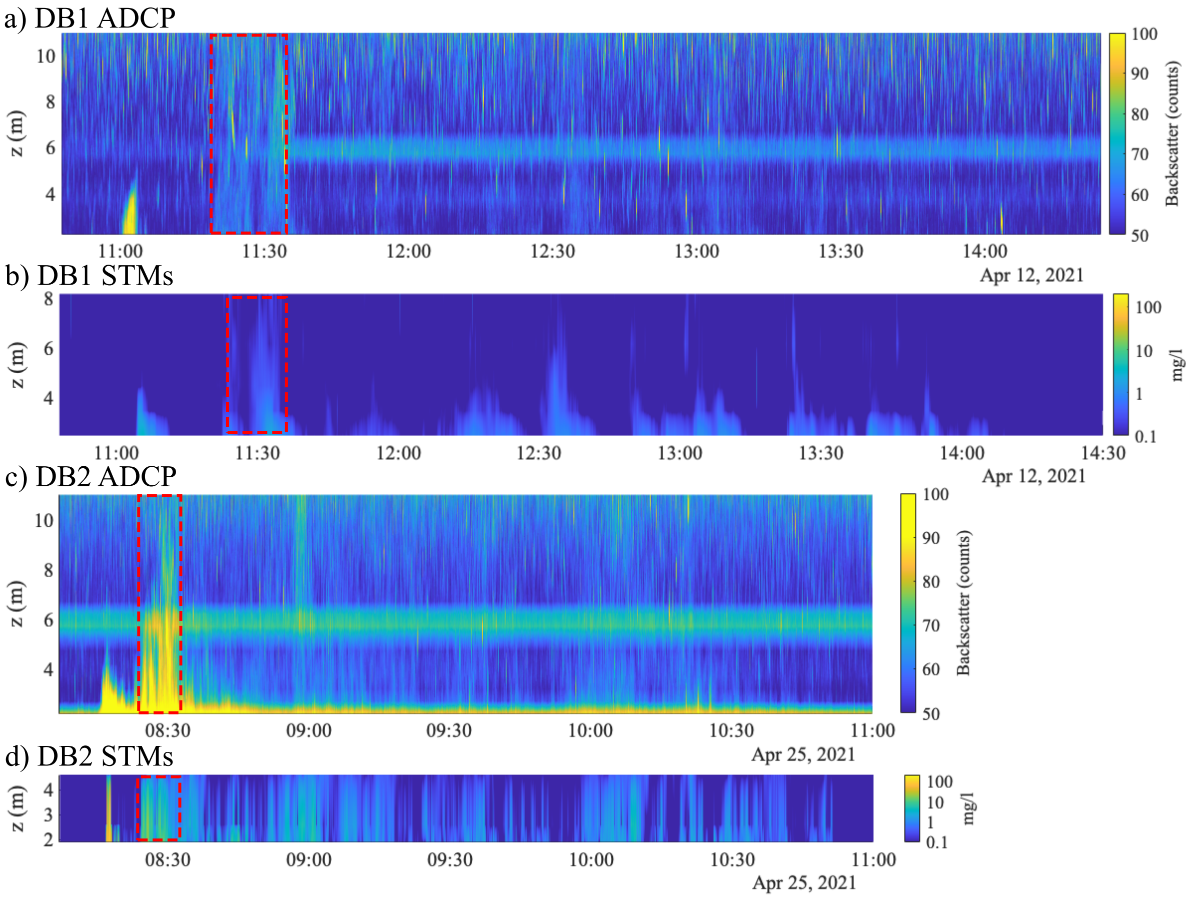

Figure S5: Vertical profiles of raw backscatter intensity and plume sediment concentration above ambient levels from the mooring-mounted down-looking 600kHz ADCP and STMs, respectively, during the a)-b) DB1 (mooring MA) and c)-d) DB2 (mooring MC) drive-by experiments. See "Drive-by experiments" section in Methods for details about the instrumentation. Both drive-by tracks were located North of the mooring, in the presence of a southward background current. At late times in the propagation of the turbidity current, the background current becomes larger than the velocity of the turbidity current front, such that even the North-propagating turbidity current front eventually becomes advected South and passes by the mooring, resulting in a continuous but finite observation time-window of the plume at the mooring. The central patch (red rectangle) of sediment is the first tall structure to pass through the mooring after the first front. In both b) and c) there is an increased level of acoustic backscatter 5-6m above the seabed due to a side-lobe reflection on the instrumented cage mounted at that height.

Local deposition observed 14 hours after selfie C2

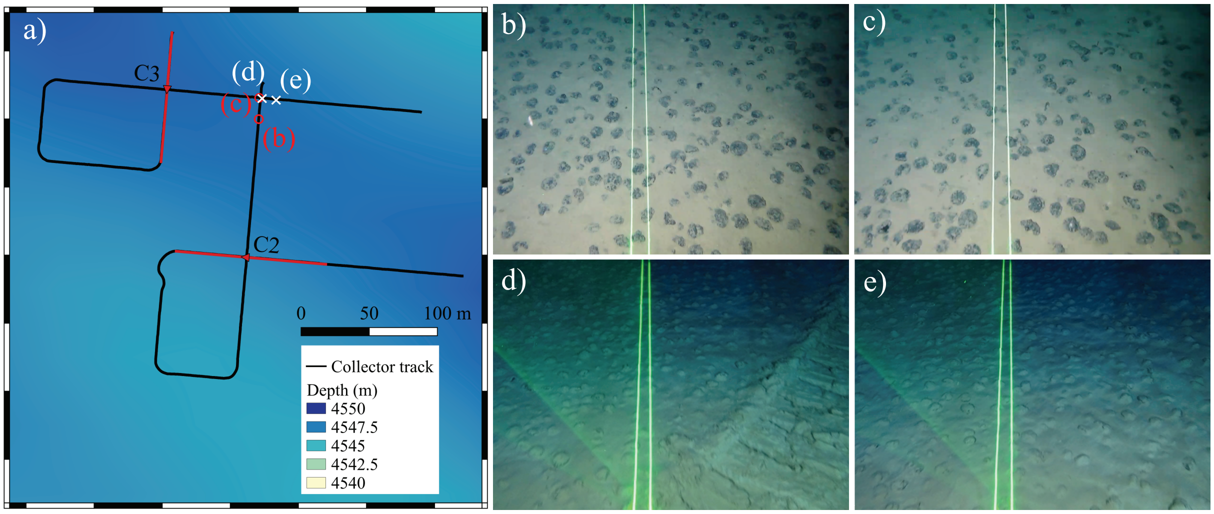

Figure S6: a) Map of the collector vehicle tracks associated with selfie experiments C2 and C3 (performed 14 hours later). (b-c) Snapshots from a front-mounted camera on the collector vehicle taken at the end of selfie C2, while the collector vehicle was driving North with the collection system turned off. Nodules were clearly visible. (d-e) Approximately 14 hours later, the same area was revisited after conducting selfie C3 with the collector vehicle driving to the East. The camera snapshots show notable blanketing of the same nodule fields as in figures (b-c) in the vicinity of the collector tracks. Indeed, on the right hand side of (d) the collector tracks from the previous day are clearly visible. The location of the collector vehicle at the time (b-e) snapshots were taken are identified in (a) with red circles (after conducting selfie C2) and white crosses (after conducting selfie C3). Note that the camera orientation in (b-c) was not the same than in (d-e). The bathymetry used in (a) was obtained from (*19*).

Seapoint Turbidity Meters calibration

The Seapoint Turbidity Meters type S (STM-S), STM-18100 to STM-18108, were mounted on the collector. These STM-S underwent two calibrations. First, the manufacturer (Seapoint) performed a factory calibration on the sensors using Formazin turbidity standard to test equipment function and sensor efficiency and stability. The aim of this first calibration is to document the instrument drift and any temporal variability in the probe's response to changes in concentration, as well as comparing the sensitivity, linearity and reproducibility of the sensors. The factory calibration consists of two points: 50 NTU (diluted from 4000NTU Formazin turbidity standard stock solution from Hach Co.) and a zero point using black tape over the windows to verify the zero output. Second, the STM-S sensors were calibrated for suspended sediment concentration (SSC) using prepared samples (diluted from 40g/L stock solution) of different concentrations of seabed CCZ sediments collected from a previous campaign in the region where the field studies were conducted. This aims at establishing a calibration relationship between the suspended sediment concentration and the turbidity signal output (in counts). This step, for the collector mounted STM-S, was conducted in laboratory prior to the field studies. During the calibration, the measurement on each sediment sample lasted for 5 minutes, in darkness, during which the sediment was maintained in suspension under constant uniform agitation with the use of a sonicator (QSonica Q500). The STM-S outputs two signals: the high range signal is more reliable for use at higher concentrations, above 1g/l. For samples with concentrations of 38mg/l and below, the low scattering signal is more reliable. The two signals overlap well in the concentration ranges between 38mg/l to 1g/l, entirely consistent with the specifications of the STM-S. During the field studies, the observed turbidity values remained within the low range values, with maximum measured concentrations of $O(100)$mg/l. As such, only the scattering signal corresponding to low sediment concentrations has been used in the results and analysis

presented in this work.

The other Seapoint Turbidity Meters are type II (STM-II), legacy analog to the STM-S, and have been used with data loggers. STM-15713 and 15714 were connected to a Seabird CTD SBE 16plus, whilst the two RBRduo logging and powering units hosted STM-13328, 15712, 15714 and 15715. The last STM was mounted on the Aquatec Aqualogger 310YT unit. The STM-II generates an output voltage proportional to the turbidity, allowing for the selection of the desired range and resolution for measurement, based on gain control, depending on the level of water turbidity. During calibration, each sensor used the same sampling frequency that was used during the field studies, as indicated in the Methods section. The calibration for these turbidity meters was conducted during the field studies aboard the vessel, following the previously detailed protocol and using the previously prepared sediment samples for the calibration of the STM-S. During all calibration events, in laboratory and aboard the vessel, special care was given to cleaning the sensor window in between measurements and experiments.

An additional Seapoint Turbidity Meter of type S, STM-18353, was brought aboard the vessel. The STM-18353 was calibrated prior to the trials, similarly to the previous all other sensors, using the same sediment samples and calibration procedure outlined in the previous paragraph. In addition to this calibration, two further calibrations on this sensor were performed. The first (in red squares in Figure S7), opted for direct weight measurement of the same sediment used for the prior calibration, rather than serial dilutions from a stock solution, using a high precision scale. The second (in blue diamonds in Figure S7), conducted aboard the vessel, used sediment samples collected during the collector trials using Niskin water sampling bottles. The samples were obtained by serial dilutions from a stock solution. The stock solution was subsequently oven dried to obtain the concentration of the suspended sediment (accounting for the mass of salt). During all three calibrations, the sediment was maintained in suspension under constant

uniform agitation with the use of a sonicator. For these three different calibrations using seabed sediment, the obtained results were entirely equivalent, as demonstrated by the data in the final panel of Figure S7.

The concentrations of the calibration samples ranged from 1mg/l to 9.8g/l. The linear calibration curve for every STM sensor used in the field studies is presented in Figure S7. The slope was obtained by minimizing $C - (a \times S)$, where C is the known concentration of the calibration sample in mg/l, a is the slope of the linear calibration curve to be determined, and S is the raw output signal from the turbidity sensor in counts. The zero output for a blank sample was verified for the turbidity sensors, using black tape over the windows and corresponded to within $\sim$ 0.1 counts of the ambient turbidity output of the STM sensors in the undisturbed water of the abyssal ocean. The concentrations reported in this study correspond to the plume sediment concentration $\hat{C} = C - C_{amb} = a \times (S - S_{amb})$, where C_{amb} and S_{amb} are the concentration and raw output from the instrument in the absence of a sediment plume, respectively, i.e. the ambient signal has been removed for each sensor. Total suspended solids concentrations in the region and depth where the studies were conducted are known to be O(0.01)mg/l (*31–33*), which is of the same order of magnitude as the sensor noise level and, therefore, it cannot be measured accurately with the STMs. Additionally, because the noise level of the STMs is $\sim$0.005 - 0.01mg/l, 0.1mg/l is chosen as the appropriate lower bound of the plume concentration logarithmic colormaps. Finally, we note that in making the calibrations presented in Figure S7, if either the "black-tape" reading for a sensor or the ambient signal of the sensor (which were in the range 0-2 counts) were used as an additional reference calibration point, assuming this corresponded to essentially zero concentration (background is expected to be around 10 $\mu g/l$, e.g. (*31–33*)), it had no notable influence ($< 0.1\%$) on the value of a for any of the calibrations.

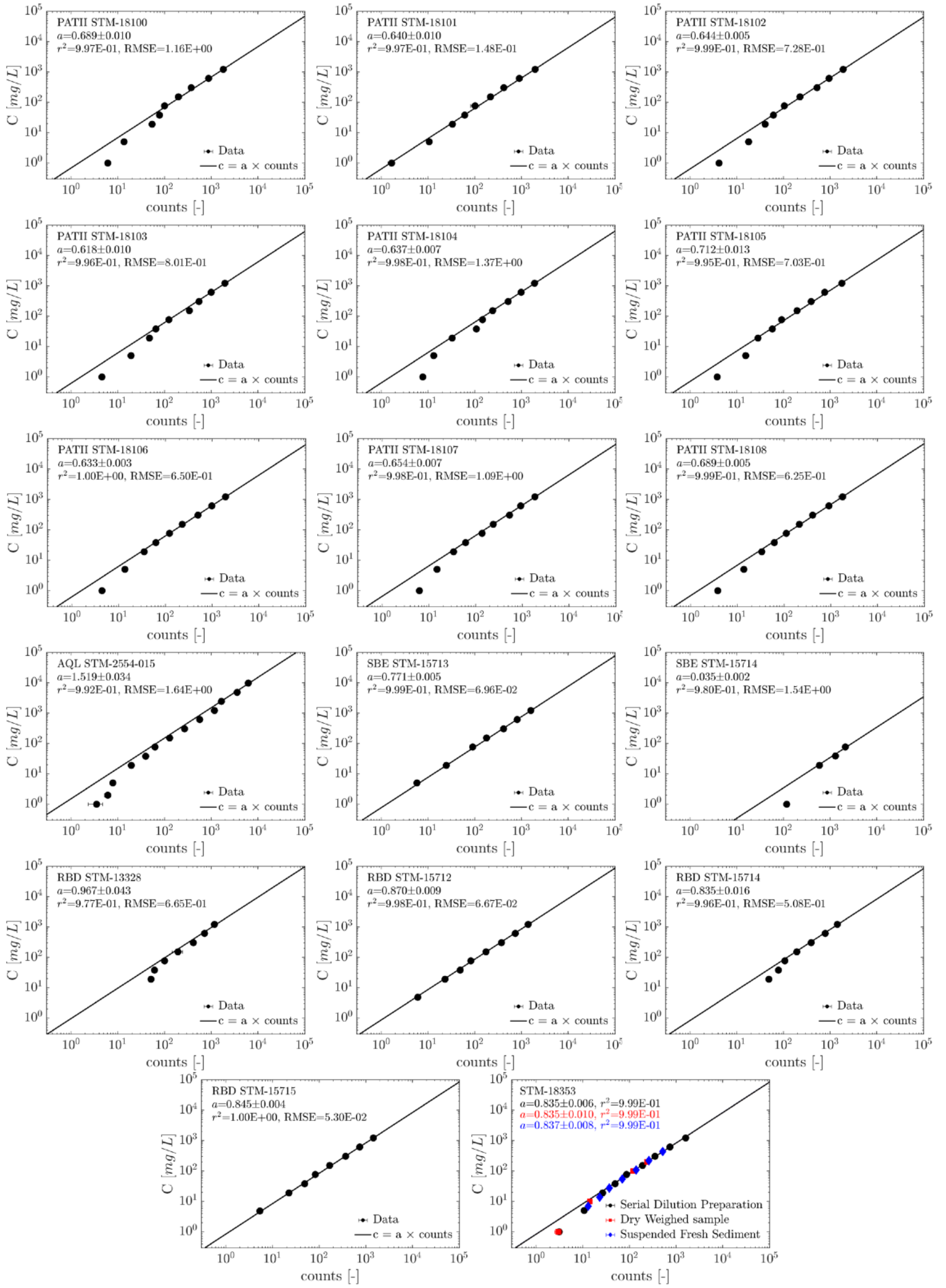

Figure S7: Calibration measurements and slope (a) of to the linear fit for each of the STM sensors used during the field studies on the collector vehicle and on the moorings. The coefficient of determination r^2 and the root mean squared error $RMSE$ the linear fits are reported.

MIX-ITOMETER calibration

The ITS MIX-ITOMETER was cross-calibrated using an STM previously calibrated in the laboratory following the procedure described in the Supplementary Material section “Seapoint Turbidity Meters calibration”. The STM was mounted on an Aquatec Aqualogger unit. For the cross-calibration, the MIX-ITOMETER was dismounted from the sediment discharge duct in the collector vehicle and submerged into seawater next to the reference STM. Seabed sediment obtained from the area of the operations was added and properly mixed with seawater. The sediment concentration was increased by adding more sediment a total of four times, until reaching a concentration of 5g/l (Figure S8a). As expected, the MIX-ITOMETER responded linearly to the sediment increase. After the field studies, ITS conducted a test to confirm the linearity of the instrument using, in this case, seawater and sand concentrations of up to 120g/l (Figure S8b). Again, the instrument responded linearly to the increase of concentration. Overall, the key outcome is that the response from the MIX-ITOMETER was linear within the range of measured concentrations during the field studies.

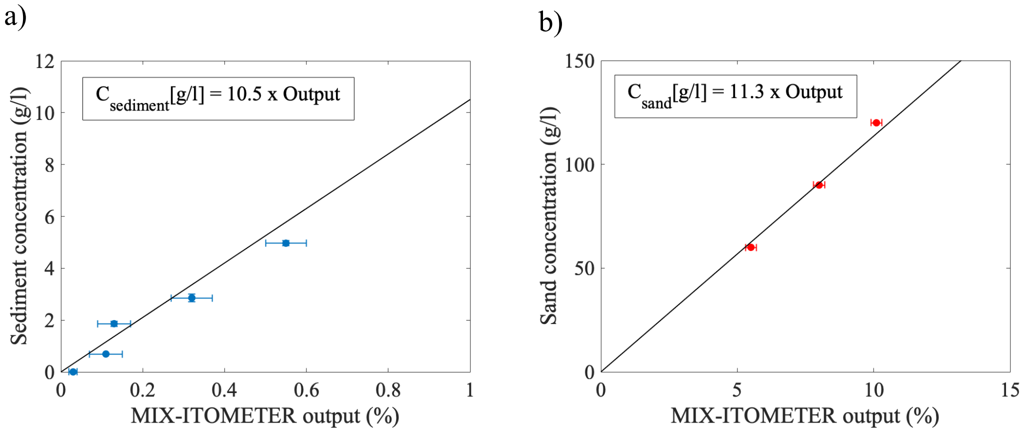

Figure S8: a) Cross-calibration curve of the MIX-ITOMETER. The blue dots correspond to the Aqualogger STM measurements and the error bars indicating the standard deviation. The red dots are reference points on the calibration curve. b) Test conducted by the instrument manufacturer to assess linear behavior of the instrument with higher concentrations using sand.

REFERENCES AND NOTES

1. T. Peacock, M. H. Alford, Is deep-sea mining worth it? *Sci. Am.* **318**, 72–77 (2018).

2. L. A. Levin, D. J. Amon, H. Lily, Challenges to the sustainability of deep-seabed mining. *Nat. Sustain.* **3**, 784–794 (2020).

3. D. Aleynik, M. E. Inall, A. Dale, A. Vink, Impact of remotely generated eddies on plume dispersion at abyssal mining sites in the Pacific. *Sci. Rep.* **7**, 16959 (2017).

4. B. Gillard, K. Purkiani, D. Chatzievangelou, A. Vink, M. H. Iversen, L. Thomsen, Physical and hydrodynamic properties of deep sea mining-generated, abyssal sediment plumes in the Clarion Clipperton Fracture Zone (eastern-central Pacific). *Elem. Sci. Anth.* **7**, 5 (2019).

5. R. E. Burns, B. H. Erickson, J. W. Lavelle, E. Ozturgut, "Observations and measurements during the monitoring of deep ocean manganese nodule mining tests in the North Pacific, March–May 1978." (Technical Report May 1978, National Oceanic and Atmospheric Administration, 1980).

6. H. Thiel, G. Schriever, A. Ahnert, H. Bluhm, C. Borowski, K. Vopel, The large-scale environmental impact experiment DISCOL—Reflection and foresight. *Deep Sea Res. Part II Top. Stud. Oceanogr.* **48**, 3869–3882 (2001).

7. F. Gausepohl, A. Hennke, T. Schoening, K. Köser, J. Greinert, Scars in the abyss: Reconstructing sequence, location and temporal change of the 78 plough tracks of the 1989 DISCOL deep-sea disturbance experiment in the Peru Basin. *Biogeosciences* **17**, 1463–1493 (2020).

8. D. D. Trueblood, E. Ozturgut, M. Pilipchuk, I. F. Gloumov, The ecological impacts of the joint US-Russian benthic impact experiment, in *Second ISOPE Ocean Mining Symposium* (International Society of Offshore and Polar Engineers (ISOPE), 1997), pp. 139–145.

9. R. E. Burns, Assessment of environmental effects of deep ocean mining of manganese nodules. *Helgoländer Meeresun.* **33**, 433–442 (1980).

10. R. Ouillon, C. Kakoutas, E. Meiburg, T. Peacock, Gravity currents from moving sources. *J. Fluid Mech.* **924**, A43 (2021).

11. H. U. Oebius, H. J. Becker, S. Rolinski, J. A. Jankowski, Parametrization and evaluation of marine environmental impacts produced by deep-sea manganese nodule mining. *Deep Sea Res. II Top. Stud. Oceanogr.* **48**, 3453–3467 (2001).

12. S. Hage, M. J. B. Cartigny, E. J. Sumner, M. A. Clare, J. E. H. Clarke, P. J. Talling, D. G. Lintern, S. M. Simmons, R. Silva Jacinto, A. J. Vellinga, J. R. Allin, M. Azpiroz-Zabala, J. A. Gales, J. L. Hizzett, J. E. Hunt, A. Mozzato, D. R. Parsons, E. L. Pope, C. D. Stacey, W. O. Symons, M. E. Vardy, C. Watts, Direct monitoring reveals initiation of turbidity currents from extremely dilute river plumes. *Geophys. Res. Lett.* **46**, 11310–11320 (2019).

13. J. W. Lavelle, E. Ozturgut, S. A. Swift, B. H. Erickson, Dispersal and resedimentation of the benthic plume from deep-sea mining operations: A model with calibration. *Mar. Mining* **3**, 59–93, (1981).

14. M. Rutkowska, K. Dubalska, G. Bajger-Nowak, P. Konieczka, J. Namieśnik, Organomercury compounds in environmental samples: Emission sources, toxicity, environmental fate, and determination. *Crit. Rev. Environ. Sci. Technol.* **44**, 638–704 (2014).

15. J. A. Jankowski, A. Malcherek, W. Zielke, Numerical modeling of suspended sediment due to deep-sea mining. *J. Geophys. Res. Oceans* **101(**C2**)**, 3545–3560 (1996).

16. Global Sea Mineral Resources, "*Environmental impact statement. Small-scale testing of nodule collector components of the seafloor of the Clarion-Clipperton Fracture Zone and its environmental impact.*" (Technical report, Global Sea Mineral Resources, 2018).

17. M. Elerian, S. Alhaddad, R. Helmons, C. van Rhee, Near-field analysis of turbidity flows generated by polymetallic nodule mining tools. *Mining* **1**, 251–278 (2021).

18. M. A. Hallworth, A. J. Hogg, H. E. Huppert, Effects of external flow on compositional and particle gravity currents. *J. Fluid Mech.* **359**, 109–142 (1998).

19. H. E. Huppert, J. E. Simpson, The slumping of gravity currents. *J. Fluid Mech.* **99**, 785–799 (1980).

20. J. O. Shin, S. B. Dalziel, P. F. Linden, Gravity currents produced by lock exchange. *J. Fluid Mech.* **521**, 1–34 (2004).

21. A. J. Hogg, M. A. Hallworth, H. E. Huppert, On gravity currents driven by constant fluxes of saline and particle-laden fluid in the presence of a uniform flow. *J. Fluid Mech.* **539**, 349–385, (2005).

22. T. C. Harris, A. J. Hogg, H. E. Huppert, Polydisperse particle-driven gravity currents. *J. Fluid Mech.* **472**, 333–371 (2002).

23. M. Wells, R. Dorrell, Turbulence processes within turbidity currents. *Annu. Rev. Fluid Mech.* **53**, 59–83 (2021).

24. S. Altinakar, W. H. Graf, E. J. Hopfinger, Weakly depositing turbidity current on a small slope. *J. Hydraul. Res.* **28**, 55–80 (1990).

25. G. V. Middleton, Sediment deposition from turbidity currents. *Annu. Rev. Earth Planet. Sci.* **21**, 89–114 (1993).

26. K. J. Curran, P. S. Hill, T. M. Schell, T. G. Milligan, D. J.W. Piper, Inferring the mass fraction of floc-deposited mud: Application to fine-grained turbidites. *Sedimentology* **51**, 927–944 (2004).

27. K. Zhao, B. Vowinckel, T. J. Hsu, T. Köllner, B. Bai, E. Meiburg. An efficient cellular flow model for cohesive particle flocculation in turbulence. *J. Fluid Mech.*, **889**, 2020.

28. J. de Leeuw, J. T. Eggenhuisen, M. J. B. Cartigny, Morphodynamics of submarine channel inception revealed by new experimental approach. *Nat. Commun.* **7**, 10886 (2016).

29. C. Muñoz-Royo, T. Peacock, M. H. Alford, J. A. Smith, A. Le Boyer, C. S. Kulkarni, P. F. J. Lermusiaux, P. J. Haley, C. Mirabito, D. Wang, E. E. Adams, R. Ouillon, A. Breugem, B. Decrop, T. Lanckriet, R. B. Supekar, A. J. Rzeznik, A. Gartman, S.-J. Ju, Extent of impact of deep-sea nodule mining midwater plumes is influenced by sediment loading, turbulence and thresholds. *Commun. Earth Environ.* **2**, 148 (2021).

30. I.-Z. Gazis, Processed EM122 multibeam swath bathymetry collected during SONNE cruise SO268/1 inside the Belgian License Area in Clarion Clipperton Zone, Pacific (2020).

31. W. D. Gardner, A. V. Mishonov, M. J. Richardson, Decadal comparisons of particulate matter in repeat transects in the Atlantic, Pacific, and Indian Ocean basins. *Geophys. Res. Lett.* **45**, 277–286 (2018).

32. W. D. Gardner, I. D. Walsh, M. J. Richardson, Biophysical forcing of particle production and distribution during a spring bloom in the North Atlantic. *Deep Sea Res. Part II* **40**, 171–195 (1993).

33. E. T. Baker, R. A. Feely, K. Takahashi, Chemical composition, size distribution and particle morphology of suspended particulate matter at DOMES sites A, B, and C: Relationships with local sediment composition, in *Marine Geology and Oceanography of the Pacific Manganese Nodule Province*, J. L Bischoff, D. Z. Piper, Eds. (Springer US, 1979), pages 163–201.
